# Geographic location and phylogeny are the main determinants of the size of the geographical range in aquatic beetles

**DOI:** 10.1186/1471-2148-11-344

**Published:** 2011-11-28

**Authors:** Pedro Abellán, Ignacio Ribera

**Affiliations:** 1 Institute of Evolutionary Biology (CSIC-UPF), Passeig Maritim de la Barceloneta 37, 08003 Barcelona, Spain; 2 Department of Bioscience, Aarhus University, Ny Munkegade 114, DK-8000 Aarhus C, Denmark

## Abstract

**Background:**

Why some species are widespread while others are very restricted geographically is one of the most basic questions in biology, although it remains largely unanswered. This is particularly the case for groups of closely related species, which often display large differences in the size of the geographical range despite sharing many other factors due to their common phylogenetic inheritance. We used ten lineages of aquatic Coleoptera from the western Palearctic to test in a comparative framework a broad set of possible determinants of range size: species' age, differences in ecological tolerance, dispersal ability and geographic location.

**Results:**

When all factors were combined in multiple regression models between 60-98% of the variance was explained by geographic location and phylogenetic signal. Maximum latitudinal and longitudinal limits were positively correlated with range size, with species at the most northern latitudes and eastern longitudes displaying the largest ranges. In lineages with lotic and lentic species, the lentic (better dispersers) display larger distributional ranges than the lotic species (worse dispersers). The size of the geographical range was also positively correlated with the extent of the biomes in which the species is found, but we did not find evidence of a clear relationship between range size and age of the species.

**Conclusions:**

Our findings show that range size of a species is shaped by an interplay of geographic and ecological factors, with a phylogenetic component affecting both of them. The understanding of the factors that determine the size and geographical location of the distributional range of species is fundamental to the study of the origin and assemblage of the current biota. Our results show that for this purpose the most relevant data may be the phylogenetic history of the species and its geographical location.

## Background

Why some species are widespread while others are very restricted geographically is one of the most basic questions in biology, although it remains basically unanswered, despite the sustained interest from ecologists, biogeographers and evolutionary biologists (e.g., [1-5]). A range of ecological and evolutionary explanations have been suggested for the observed range size variation, based on differences in niche breadth or environmental tolerance, body size, population abundance, latitude, environmental variability, colonization and extinction dynamics, and dispersal ability [3,6-8]. However, there are still fundamental questions unresolved, best exemplified by the fact that closely related species often display dramatic differences in range size for largely unknown reasons. Tests of these differences remain relatively scarce, have been performed for examples of very few taxa (usually vertebrates), and generally fail to address the potentially confounding effects of the phylogenetic relatedness of species.

In this work we aim to test some likely determinants of the size of the geographical range in a phylogenetic comparative framework. Closely related species are expected to show more similarity than those that are distantly related because they share more common evolutionary history [9,10]. How range size evolves and the extent of heritability of the geographical range sizes of species has received much attention in the last years [11-14], as evidence for range size heritability would have important implications for ecology, evolution, and biogeography [15]. Although a number of studies have investigated the existence of phylogenetic signal in range size in a variety of clades and from a wide range of analytical approaches, the patterns found have been mixed and the "heritability" of range size remains a contentious issue [13,14]. Phylogenetic comparative methods applied to whole lineages and not only species-pairs (e.g., [16-18]) may provide a more robust and powerful approach to estimate the phylogenetic signal in range size.

Among the potential determinants of range size we include ecological tolerance, dispersal ability, geographic location, and age of the species. For all of them we test their phylogenetic signal, as well as the phylogenetic signal of the size and location of the range itself.

1) Ecological tolerance. A broad ecological niche allows a species to persist in a wide range of different environments, while a narrow niche restricts a species to the few places where its niche requirements are met [19,20]. Hence, species with broad niches should be distributed over a wider range of different biomes than species with narrower ecological requirements, leading to larger geographical ranges [21]. When these biomes are distributed equally along environmental gradients this poses the problem that species with lager ranges will inevitably overlap more different biomes. However, in the western Palaearctic (the centre of distribution of most of our studied lineages, see below) this problem is partly alleviated by the very heterogeneous distribution of environmental gradients. In this case, those species occurring in large biomes (e.g., [20,21]) would have large range sizes (as found e.g. by [14]), but they should not necessarily occupy more biomes than species with smaller ranges.

2) Dispersal ability. As a surrogate measure of dispersal ability we use water flow, as previous studies in freshwater invertebrates have established the relationship between main habitat type (lotic or lentic) and the size of the geographical range [22,23]. Lentic species should have better dispersal abilities due to the shorter geological duration of their habitats, and display on average larger geographical ranges than the species inhabiting the more persistent lotic habitats [24]. However, the role of habitat constraints in aquatic organisms has yet not been assessed from a phylogenetic comparative framework in lineages in which there are species inhabiting both habitat types.

3) Geographic location. There are multiple cases of closely related species with a similar biology and ecology with extreme differences in the size of the geographical range. In these cases, the biogeographic settings in which species arise and evolve could determine their range sizes [14,25], with species with a "privileged" geographical position displaying higher range-sizes. For example, latitudinal gradients in geographic range size (Rapoport's rule) have been extensively studied and documented [6,26,27]. Evidence supporting that range sizes increase with latitude in the Palearctic and Nearctic above 40°-50°N has been found in a number of terrestrial groups [26], but the extent to which this is a general pattern remains contentious and has rarely been tested in a phylogenetic framework.

4) Age and area. We also consider the possible relationship between age and area, which requires to be tested in the context of a phylogeny even if not in the same comparative framework as the previous factors. Originally proposed to explain the distribution of the endemic flora of some islands [28], in its basic form the "age and area" hypothesis states that the older a species is the more likely it is to have occupied a wider geographical area. More precisely, it could be expected that species have a "life cycle" from origin to extinction that could be described through a variety of simple models (see [26] for a review). Although a number of studies have examined the evidence for geographic range size changes over evolutionary time across a wide range of clades (but never in insects), no consistent evidence has emerged to support any particular model [29]. An inherent limitation of all these studies is that a direct test of the age and area model requires the geographic range size of a species or a clade to be known throughout its evolutionary history [3]. This is usually not possible without extensive palaeontological data. Hence, a different approach has to be taken in neontological studies, considering interspecific variation in range sizes of contemporary species as a reflection of the intraspecific relationship [30]. The examination of the interspecies relationship between geographic range size and age could thus be used as a surrogate of the transformation of range size with age in individual species [29,31].

We use a set of lineages of closely related species of different families of aquatic Coleoptera to investigate the relative role of different factors on determining the size of the geographical range of species. The Western Palearctic water beetle fauna is a suitable model to study range size issues, as water beetles are a rich and well-known insect group in both Europe and the Mediterranean Basin, exhibiting a high level of endemism but also with species widely distributed across the Palearctic and Holartic regions [32-34]. Spatial determinants of range size and temporal patterns of range evolution in invertebrates may differ substantially from that found in previous studies using vertebrate clades. Hence, the use of phylogenies at the species level for different groups of beetles, one of the most diverse and understudied lineages of animals, in what is in fact a set of independent evolutionary replicates, provides a unique opportunity to assess these issues from a phylogenetic comparative framework.

## Methods

### Background on the studied groups

We have used a phylogenetically heterogeneous set of ten monophyletic lineages of water beetles (Table 1) occurring in the western Palearctic, some with both lotic and lentic species, and others encompassing exclusively either lotic or lentic species. The lineages used here belong to three different families of two suborders of Coleoptera (Adephaga and Polyphaga), representing several independent invasions of the aquatic medium [35]. The full list of species and data used in this study are provided in Additional file 1.

**Table 1:** Lineages of water beetles studied.

Lineage	Taxa	Habitat
*Ilybius subaeneus *group (Dytiscidae)	27 (33)	Lentic
Western Mediterranean *Deronectes *(Dytiscidae)	24 (29)	Lotic
Subgenus *Enicocerus *(Hydraenidae)	9 (14)	Lotic
*Limnebius nitidus *subgroup (Hydraenidae)	10 (10)	Lotic
*Hydraena gracilis *lineage (Hydraenidae)	14 (27)	Lotic
*Hydraena dentipes *lineage (Hydraenidae)	20 (28)	Lotic
"Phothydraena" lineage (Hydraenidae)	9 (9)	Lotic
Palaearctic *Graptodytes *(Dytiscidae)	18 (23)	Mixed
*Hydroporus planus *group s.l. (Dytiscidae)	30 (52)	Mixed
West Palaearctic *Hydrochus *(Hydrochidae)	13 (14)	Mixed

Lineages of water beetles included in this study. The number of species included (in parenthesis the total number of species in the lineage) and the habitat preference are indicated.

#### Family Dytiscidae

1) The *Ilybius subaeneus *group (genus *Ilybius *[36]) includes 33 recognized species, occurring almost exclusively in stagnant water and with generally wide geographical ranges throughout large parts of the Palearctic or Nearctic, with some Holarctic species [36,37]. Together with the genus *Rhantus*, they are the most species-rich clade of the Palearctic fauna confined to stagnant water. Our dataset included 27 species.

2) The genus *Deronectes *is the largest clade of Palearctic Dytiscidae entirely confined to running waters, with a predominantly Mediterranean distribution reaching central Asia in the east [36]. Species are usually restricted to relatively small geographical ranges, frequently in mountain regions. Here, we focused on the western Mediterranean clade, encompassing 26 recognized species or subspecies [38] of which our final dataset included 24.

3) The genus *Graptodytes *includes 21 recognized species distributed in the western Palearctic region [39], with both lotic and lentic species. Our final dataset included 18 taxa.

4) The *Hydroporus planus *group (genus *Hydroporus*) includes 51 species with a Palearctic distribution [36,40], also with both lotic and lentic species. We sampled 30 species, including most of the western Palearctic fauna.

#### Family Hydraenidae

5) The subgenus *Enicocerus *(genus *Ochthebius*) includes 15 recognized species exclusive of running waters [41], distributed in Europe and the middle East. We studied 9 species, including all member of the *O. exculptus *group [41].

6) The genus *Limnebius *Leach, with an almost world-wide distribution [42], is one of the most diverse genera of the family Hydraenidae. In his revision of the Palearctic species, Jäch [42] recognized several species groups, based on both external morphology and the structure of the male genitalia. Among them, the *Limnebius nitidus *subgroup includes 11 western Palearctic species with a rather uniform external morphology [43]. Several species of this lineage have very restricted allopatric distributions, often limited to a single valley or mountain system, but there are also some species with wider geographical ranges. All inhabit running waters. We included all the species within this subgroup with a sole exception (*L. nitifarus*).

7-8) The "*Haenydra*" lineage (genus *Hydraena*) currently includes 86 recognized species [44,45] usually found in clean, fast flowing waters, often in mountain streams. They are distributed in the north Mediterranean region from Iberia to Iran. Many species of this lineage have very restricted distributions, often limited to a single valley or mountain system, but there are also some species with very wide geographical ranges, such as e.g., *H. gracilis*, present in the whole Europe from north Iberia to the Urals [45]. Here, we included two different monophyletic lineages within "*Haenydra*": the *H. gracilis *and the *H. dentipes *clades [46], with 27 and 28 species respectively, of which we include 14 and 20.

9) The "*Phothydraena" *lineage (genus *Hydraena *[47]) currently include 9 recognized species, usually found in clean, fast flowing waters, often in mountain streams. Molecular data were available for seven species.

#### Family Hydrochidae

10) The genus *Hydrochus *includes about 180 described species [48]. In the west Mediterranean (Iberian Peninsula, Morocco and south France) the genus is represented by 12 recognized species, 7 of them endemic to the area, which form a monophyletic group that also includes *H. roberti*, so far recorded from the Caucasus and Turkey [49]. We include 12 of the 13 species of this clade.

### Phylogenetic data

We reconstructed the phylogenetic relationships within each lineage of water beetles from different combinations of mitochondrial and nuclear genes, depending on data availability. Phylogenies of *Graptodytes*, *Enicocerus*, "*Haenydra*" and *Hydrochus *were taken from recent works ([39,41,46,49]) respectively), pruning the trees to keep one specimen per species. Phylogenies of *Deronectes*, *Ilybius *and *Hydroporus *were updated with additional species and new analyses from [38] and [40] respectively; finally, phylogenies of *Limnebius *and *Phothydraena *were newly built for this work, with the same genes and methodology used in [46] for the two lineages of "*Haenydra*" (see Additional file 1 for details of the sequence data used for each lineage, Additional file 2, Table S1 for the primers used for amplification and sequencing, and Additional file 3 for the final trees used).

New phylogenies were built using a fast maximum likelihood algorithm as implemented in RAxML v7.0 [50], after aligning length-variable regions with MAFFT v5.8 [51]. For the RAxML searches we used a partition by gene fragment, with a GTR+G evolutionary model independently estimated for each partition, following the methodology used in [46]. To estimate the relative age of divergence of the lineages we used the Bayesian relaxed phylogenetic approach implemented in BEAST v1.4.7 [52], which allows variation in substitution rates among branches. We implemented a GTR+I+G model of DNA substitution with four rate categories using the mitochondrial data set, as the *a priori *rate used was estimated for mitochondrial genes only (see below). We used an uncorrelated lognormal relaxed molecular clock model to estimate substitution rates and the Yule process of speciation as the tree prior. Well supported nodes in the analyses of the combined sequence (when nuclear genes were used) were constrained to ensure that the Beast analyses obtained the same topology. We ran two independent analyses for each group sampling each 1000 generations, and used TRACER version 1.4 to determine convergence, measure the effective sample size of each parameter and calculate the mean and 95% highest posterior density interval for divergence times. Results of the two runs were combined with LogCombiner v1.4.7 and the consensus tree compiled with TreeAnnotator v1.4.7 [52]. As each lineage was analysed separately, to establish the relationship between age and size of the geographical range we only require a relative dating of species within the lineage, not an absolute dating. Notwithstanding this, we used an approximate dating using as prior evolutionary rate for the combined mitochondrial sequence (including protein coding and ribosomal genes) a normal distribution with average rate of 0.01 substitutions/site/MY, with a standard deviation of 0.001. This rate is close to recent estimations of different groups of Coleoptera [46,53] and to the standard arthropod mitochondrial clock of 2.3% [54,55].

The evolutionary age of each species was calculated as the estimated age (in millions of years) of the most recent node that connects it to any other taxon or clade. The age estimates of Beast have usually large 95% confidence intervals, which has to be considered in the interpretation of the Results.

### Geographical data and biogeographic factors

We created shaded maps of the distribution of the different species in a Geographic Information System based on the information compiled from published and unpublished sources [36,37,44,48,56,57]; checklist of the species of the Italian fauna, v. 2.0, http://www.faunaitalia.it). This resulted in individual species maps containing one or more polygons of distribution (species maps are available from the authors upon request). We then calculated different descriptors of the species' ranges: total range-size, maximum latitudinal and longitudinal limits, and latitudinal and longitudinal centroids.

Total range-size was calculated as the total area of the polygon or polygons, after reprojecting species maps to equal-area projections. For those species only known from their locality type (four species), range size was arbitrarily set to 100 km^2^. Latitudinal and longitudinal centroid positions (centre of mass of the polygon or polygons) and maximum latitudinal and longitudinal limits were computed as geographical coordinates.

All spatial data were processed using ArcGIS 9.2 software (Environmental Systems Research Institute Inc., Redlands, CA). Area variables (total range-size and average size of biomes) were log10 transformed for the analyses.

### Ecological factors

The main habitat type of the studied species was defined according to the general water flow regime, and three categories were distinguished: (1) lotic (strictly running water); (2) both running and standing water; and (3) lentic (strictly standing water) (see [22] for details on habitat choice criteria) (Additional file 1). For some analyses we pooled species in categories (2) and (3), thus dividing species limited to running water from the rest. Water flow is the most important habitat characteristic determining the composition of the assemblages of aquatic Coleoptera, and species tend to be restricted to either standing water bodies or to running water, both in the larval and in the more dispersive adult stage (see [22,24] and references therein).

To determine the role of niche breadth on range-size we used the number of different biomes that partly or completely overlap with the species ranges based on the biomes delineated by the World Wildlife Fund (http://www.worldwildlife.org/science/ecoregions/item1847.html). We discarded biomes that overlapped less than 1% of the species' range, to minimize the effects of uncertainty in range size calculations. To try to disentangle the effect of the range size *per se *from that of an increased ecological tolerance, we tested whether species that are found in larger biomes have larger ranges than species in smaller biogeographic units. If so, this would suggest that range size is a function of the available biogeographic space, rather than the number of biomes being a function of the size of the range. For that purpose we calculated the mean size of the biomes that partly or completely overlap with the species ranges [25].

### Data analyses

#### Phylogenetic signal

We used a randomization procedure to test whether range attributes exhibit a significant tendency for related species to resemble each other according to the methodology proposed by Blomberg *et al*. [17]. The basic idea is to ask whether a given tree (topology and branch lengths) better fits a set of tip data as compared with the fit obtained when the data have been randomly permuted across the tips of the tree, thus destroying any phylogenetic signal that may have existed [17]. Thus, the degree of resemblance among relatives can be distinguished from random by comparing observed patterns of the variance of independent contrasts of the trait to a null model of shuffling taxa labels across the tips of the phylogeny.

To quantify the amount of phylogenetic signal we calculated the metric *K*, which compares the observed signal in a trait to the signal under a Brownian motion model of trait evolution on a phylogeny [17]. The higher the *K *statistic, the more phylogenetic signal in a trait. *K *values of 1 correspond to a Brownian motion process, which implies some degree of phylogenetic signal. *K *values closer to zero correspond to a random or convergent pattern of evolution, while *K *values greater than 1 indicate strong phylogenetic signal. We used the R package 'Picante' [58] to compute *K *and the significance test.

We also used phylogenetic eigenvector regression (PVR; [59]) as an additional assessment of the phylogenetic signal in range properties and to correct for this signal in analysing the relationship between range-size and biogeographical variables (see below). The basic idea of PVR is to carry out a principal coordinate analysis of the matrix of pairwise phylogenetic distances between species and use the eigenvectors as predictors in a multiple regression against species traits (in this case, geographic range properties). The subset of eigenvectors to use as PVR components for each range attribute was obtained using a stepwise multiple regression [60]. The *R*^2 ^of the multiple regression model of the trait against the eigenvectors provides an estimate of the amount of phylogenetic signal in the data [59].

To assess if habitat occupation exhibited phylogenetic signal, habitat type was used as a qualitative (discretely-coded) character, and species were assigned to either of the three habitat type classes: lotic, lentic or both. In this case, phylogenetic signal was computed with Pagel's λ [16,18], a more appropriate approach for discrete traits. The value of λ varies from 0 to 1, where 0 corresponds with the complete absence of phylogenetic structure and 1 means that variation in the trait is perfectly correlated with phylogeny. We used the fitDiscrete function of the R package 'Geiger' [61] to obtain the maximum likelihood estimate of λ. In order to address whether significant phylogenetic signal existed in our datasets, we compared the negative log likelihood when there was no signal (i.e. using the tree transformed lambda = 0) to that when lambda was estimated using the original tree topology by using a likelihood ratio test.

#### PGLS correlations

We explored the association between range size and different biogeographical and ecological factors in a phylogenetic framework. We used the Phylogenetic Generalized Least Squares approach (PGLS; [62]) as implemented in Compare 4.6 b, which allows tests for correlations between two continuous traits and between a discrete independent variable and a continuous dependent variable [63]. PGLS can be viewed as an extension of Felsenstein's independent contrasts method [64] that allows for flexibility in the underlying evolutionary assumptions. This flexibility is obtained through the use of a single parameter (alpha), which can be interpreted as a measure of evolutionary constraint acting on the phenotypes. When alpha is small, generalized least squares approximates Felsenstein's independent contrasts analysis, and when alpha is large, comparative data are less dependent on phylogeny and approximate a raw, nonphylogenetic correlation analysis. The Compare software computes the maximum likelihood estimate of alpha (from a range of different alphas), and provides parameter estimates given that maximum likelihood. To assess the significance of the relationship between traits we tested if the regression slope differed from zero. Since the correlation coefficient is directly related to the regression slope, if this differs significantly from zero, the correlation coefficient will too. For this, we used the corMartins function of the R package 'Ape' [65] with the estimated value of alpha to create the correlation structure, and then fitted the linear model with the gls function.

#### Range-size *vs*. Age

In order to examine the relationship between geographic range size and species age, plots of range size (log10 transformed) against species age (i.e. the estimated age of divergence between species) were produced for each group [29,31]. Statistical significance was determined using linear or quadratic regressions as appropriate according to an extra sum-of-squares F test.

#### Global determinants of range size

A multiple regression analysis was used to determine the relative importance of the different variables, regressing the log-transformed range-size (response variable) upon the explanatory variables (range properties, species' age, niche breadth and habitat preference) and the phylogenetic PVR components. Habitat type was coded as a dummy variable (0, strictly lotic species; 1, species inhabiting lentic waters or both lentic and lotic waters).

Preliminary analyses showed that some subsets of the geographic properties of the range were often correlated. This was also corroborated by visual examination through Principal Component Analysis. Similarly, the average size of biomes and the number of biomes were usually highly correlated with maximum latitude or longitude. As a consequence, high levels of multicollinearity were detected, as indicated by high values of the Variance Inflation Factor [66]. To avoid this multicollinearity, only maximum latitude and longitude (the main determinants of range-size as assessed by PGLS, and usually not correlated between them) were finally used as biogeographic factors. In those lineages in which both variables (maxLat and maxLon) were significantly correlated, only the main determinant of range size was used.

We used a stepwise model selection procedure to select the multiple linear regression models with the smallest Akaike's Information Criterion (AIC). To account for multiple comparisons we applied the Bonferroni correction.

## Results

### Phylogenetic signal

Range size showed significant phylogenetic signal (as measured with a randomization test) in four of the ten lineages, displaying relatively low values of the *K *statistic (Table 2). However, no phylogenetic signal remained statistically significant for range size after Bonferroni correction. The maximum longitude and the longitude of centroid showed significant phylogenetic signal for most of the studied lineages (the exceptions were *Ilybius*, *Enicocerus *and *Hydrochus*, the former two encompassing few taxa) with values of *K *generally high, while minimum longitude, maximum latitude, and the latitude of centroid showed significant phylogenetic signal in four of the lineages. After Bonferroni correction, only some lineages showed significant phylogenetic signal, with maximum longitude displaying the higher number of significant cases.

**Table 2:** Phylogenetic signal (*K *statistic)

Lineage	Size	maxLon	minLon	maxLat	minLat	LonC	LatC	avB	nB
*Ilybius*	0.06 (0.787)	0.24 (0.076)	0.11 (0.456)	0.13 (0.418)	0.16 (0.201)	0.16 (0.189)	0.23 (0.097)	0.07 (0.729)	0.11 (0.565)
*Deronectes*	0.22 (0.386)	0.91 (0.001*)	0.63 (0.038*)	0.19 (0.581)	0.41 (0.037*)	0.91 (0.003**)	0.26 (0.262)	0.25 (0.228)	0.31 (0.215)
*Enicocerus*	0.48 (0.777)	0.60 (0.572)	0.65 (0.525)	0.30 (0.977)	0.74 (0.547)	0.59 (0.613)	0.32 (0.972)	0.42 (0.861)	0.42 (0.923)
*Limnebius*	0.76 (0.034*)	1.06 (0.042*)	0.73 (0.041*)	1.23 (0.003**)	0.43 (0.315)	0.90 (0.046*)	1.00 (0.009*)	0.47 (0.186)	0.72 (0.071)
*H. gracilis*	0.59 (0.052)	0.829 (0.011*)	0.329 (0.515)	0.28 (0.655)	0.69 (0.017*)	0.69 (0.018*)	0.26 (0.730)	0.22 (0.836)	0.54 (0.111)
*H. dentipes*	0.60 (0.046*)	1.30 (0.000**)	0.73 (0.008*)	1.11 (0.000**)	0.21 (0.690)	1.22 (0.000**)	0.78 (0.008*)	0.50 (0.086)	0.82 (0.013*)
*Phothydraena*	0.16 (0.841)	0.70 (0.039*)	0.85 (0.046*)	0.33 (0.542)	0.49 (0.243)	1.08 (0.005**)	0.74 (0.105)	0.63 (0.059)	0.20 (0.668)
*Graptodytes*	0.30 (0.019*)	0.62 (0.001**)	0.27 (0.069)	0.39 (0.008*)	0.22 (0.067)	0.55 (0.000**)	0.65 (0.000**)	0.19 (0.099)	0.33 (0.009*)
*Hydroporus*	0.57 (0.008*)	0.81 (0.001**)	0.31 (0.318)	0.62 (0.018*)	0.64 (0.022*)	0.69 (0.003**)	0.92 (0.001**)	0.36 (0.289)	0.32 (0.119)
*Hydrochus*	0.73 (0.394)	0.88 (0.173)	0.80 (0.45)	0.81 (0.258)	0.62 (0.617)	0.87 (0.192)	0.82 (0.226)	0.40 (0.898)	0.72 (0.375)

Phylogenetic signal as estimated with *K *statistic for different range attributes. The *P*-value, based on the variance of phylogenetically independent contrasts relative to tip shuffling randomization, is provided in parenthesis. Asterisks indicate significant *P*-values: * *P *< 0.05; ** *P *< 0.005 (Bonferroni critical value for ten tests). Codes: Size, range size (log-transformed); maxLong and minLong, maximum and minimum longitude of ranges, respectively; maxLat and minLat, maximum and minimum latitude, respectively; LonC and LatC, longitude and latitude of centroids, respectively; avB, average size of the biogeographic provinces that partly or completely overlap with the species ranges (log-transformed); nB, number of biogeographic provinces that partly or completely overlap with the species ranges.

The average size of biomes did not show phylogenetic signal in any lineage whereas niche breadth, as estimated by number of biomes overlapping with geographic range, displayed phylogenetic signal only for the *H. dentipes *and *Graptodytes *lineages. Three lineages did not exhibit phylogenetic signal for any of the range attributes: *Ilybius*, *Enicocerus *and *Hydrochus*.

The phylogenetic eigenvector regression (PVR) gave in general a stronger phylogenetic signal than the randomization tests. This was especially evident in *Ilybius*, for which PVR showed high levels of phylogenetic signal in contrast with non significant *K *values. Range-size showed moderate levels of phylogenetic signal, but some descriptors of the spatial position of ranges had higher levels (Table 3), which remained mostly significant after Bonferroni correction. Notably, a high fraction of the variance in the northern longitudinal limits and longitudinal centroids of the ranges in most lineages was explained by phylogenetic relationships among species. As happened with the randomization tests, the average size of biomes and the number of biomes were rarely correlated with phylogeny (Table 3).

**Table 3:** PVR coefficients

Lineage	Size	maxLon	minLon	maxLat	minLat	LonC	LatC	avB	nB
*Ilybius*	0.843**	0.983**	1.000**	0.719**	0.651**	0.862**	0.222	0.103	0.904**
*Deronectes*	0.148	0.981**	0.343*	0.329*	0.470**	0.953**	0.462*	0.211*	0.160*
*Enicocerus*	0.475*	0.401	0.425	0.858**	0.413	0.507*	0.640*	0.316	0.301
*Limnebius*	0.457*	0.968**	0.766*	0.936**	0.681*	0.932**	0.458*	1.000**	0.358
*H. gracilis*	0.581*	0.990**	0.558*	0.601*	0.367*	0.256	0.786**	0.236	0.564*
*H. dentipes*	0.668**	0.950**	0.948**	0.631**	0.206**	0.946**	0.389**	0.883**	0.391*
*Phothydraena*	0.550*	0.743*	0.599*	0.344	0.480*	0.984**	0.870**	0.411*	0.537*
*Graptodytes*	0.567*	0.897*	0.644**	0.427*	0.185	0.687**	0.975**	0.492**	0.702*
*Hydroporus*	0.943**	0.737**	0.910**	0.979**	0.688**	0.709**	0.747**	0.852**	0.402*
*Hydrochus*	0.489	0.371*	0.987**	0.208	0.238	0.822**	0.415*	0.236	0.270

Coefficients of determination of Phylogenetic Eigenvector Regression (PVR) models for the different lineages studied. Variable codes as in Table 2. Size, range size (log-transformed). Models with significant *P*-values are indicated with asterisks: * *P *< 0.05; ** *P *< 0.005 (Bonferroni critical value for ten tests).

Among the lineages with species with both habitat types (lotic and lentic), habitat type exhibited significant phylogenetic signal for *Hydroporus*, as computed with Pagel's λ (λ = 0.89, *P *< 0.01), but not for "*Phothydraena*" (λ < 0.00, *P *= 1.0), *Graptodytes *(λ < 0.00, *P *= 0.4) or *Hydrochus *(λ = 1, *P *= 0.3). Estimates of the phylogenetic signal with the *K *statistic gave similar results, with *Hydroporus *exhibiting significant signal (*K *= 0.63, *P *< 0.01) but not the remaining lineages (*Phothydraena*, *K *= 0.17, *P *= 0.9; *Graptodytes*, *K *= 0.15, *P *= 0.3; *Hydrochus*, *K *= 0.98, *P *= 0.1).

### Ecological tolerance

Range size was significantly and positively correlated with both the spatial extent and the number of biomes in which species are found in most of the tested lineages, as measured after phylogenetic correction with PGLS correlations (Table 4). Correlations remained significant after Bonferroni correction only in the case of the number of occupied biomes.

**Table 4:** PGLS tests for associations between range-size and biogeographical and ecological variables

		Variables								
Lineage		maxLon	minLon	maxLat	minLat	LonC	LatC	avB	nB	Hab
*Ilybius*	α	15.5	15.5	15.5	15.5	15.5	15.5	15.5	15.5	
	r	0.08	-0.50	0.74	-0.26	-0.27	0.50	0.18	0.67	--
	*P*	0.888	0.010*	0.000**	0.317	0.133	0.015*	0.378	0.000**	
*Deronectes*	α	15.5	15.5	13.95	15.5	15.5	15.5	15.5	15.5	
	r	0.43	-0.06	0.74	-0.32	0.22	0.29	0.62	0.81	--
	*P*	0.027*	0.974	0.000**	0.164	0.224	0.183	0.001**	0.000**	
*Enicocerus*	α	15.5	15.5	15.5	15.5	15.5	15.5	4.6	4.66	
	r	0.86	0.59	0.88	-0.02	0.8	0.78	0.75	0.91	--
	*P*	0.003**	0.096	0.002**	0.955	0.01	0.014*	0.026*	0.000**	
*Limnebius*	α	4.16	2.15	4.99	1.10	2.89	4.50	2.23	10.62	
	r	0.60	-0.56	0.62	-0.54	0.20	0.41	0.74	0.89	--
	*P*	0.08	0.091	0.069	0.092	0.641	0.298	0.013*	0.000**	
*H. gracilis*	α	9.91	1.86	2.89	3.56	4.53	2.01	11.7	15.5	
	r	0.67	-0.32	0.66	-0.28	0.35	0.53	0.55	0.67	--
	*P*	0.006*	0.513	0.007*	0.181	0.111	0.067	0.032*	0.014*	
*H. dentipes*	α	15.5	2.86	15.5	2.29	15.5	15.5	3.5	5.37	
	r	0.84	0.01	0.84	0.15	0.64	0.71	0.53	0.71	--
	*P*	0.000**	0.395	0.000**	0.717	0.002**	0.000**	0.014*	0.000**	
*Phothydraena*	α	15.5	15.5	15.5	15.5	15.5	15.5	15.5	15.5	14.59
	r	0.29	-0.17	0.42	-0.60	0.06	0.08	0.23	0.38	0.76
	*P*	0.494	0.65	0.269	0.095	0.919	0.84	0.607	0.353	0.019*
*Graptodytes*	α	15.5	1.87	7.66	15.5	15.5	15.5	3.41	15.5	3.64
	r	0.74	-0.43	0.85	0.301	0.525	0.79	0.09	0.67	0.84
	*P*	0.000**	0.579	0.000**	0.156	0.017*	0.000**	0.847	0.014*	0.000**
*Hydroporus*	α	2.33	2.15	3.18	1.95	4.98	6.56	3.35	4.08	5.36
	r	0.75	-0.32	0.86	-0.16	0.5	0.74	0.61	0.84	0.53
	*P*	0.000**	0.3013	0.000**	0.433	0.001**	0.000**	0.000**	0.000**	0.001**
*Hydrochus*	α	3.50	4.36	15.5	2.66	4.32	15.5	15.5	15.5	3.71
	r	0.69	-0.06	0.78	-0.61	0.39	0.49	0.57	0.65	0.27
	*P*	0.010*	0.859	0.002*	0.034*	0.185	0.091	0.041*	0.015*	0.398

Results of phylogenetic comparative tests for associations between range-size and biogeographical and ecological variables using Phylogenetic Generalized Least Squares. The maximum likelihood estimate of evolutionary constraint (alpha, α) is provided, and parameter estimates given that maximum likelihood. Significant relations (regression slope significantly different of zero) are given in asterisks: * *P *< 0.05; ** *P *< 0.005 (Bonferroni critical value for ten tests).

### Dispersal ability

Habitat preference, taken as a surrogate of dispersal ability, was significantly and positively correlated with range-size after phylogenetic correction with PGLS in those lineages with both lotic and lentic species, with the only exception of *Hydrochus *(Table 4). Correlation values were particularly high for *Phothydraena *and *Graptodytes*, which remained significant after Bonferroni correction.

### Geographic location

**Figure 1:**
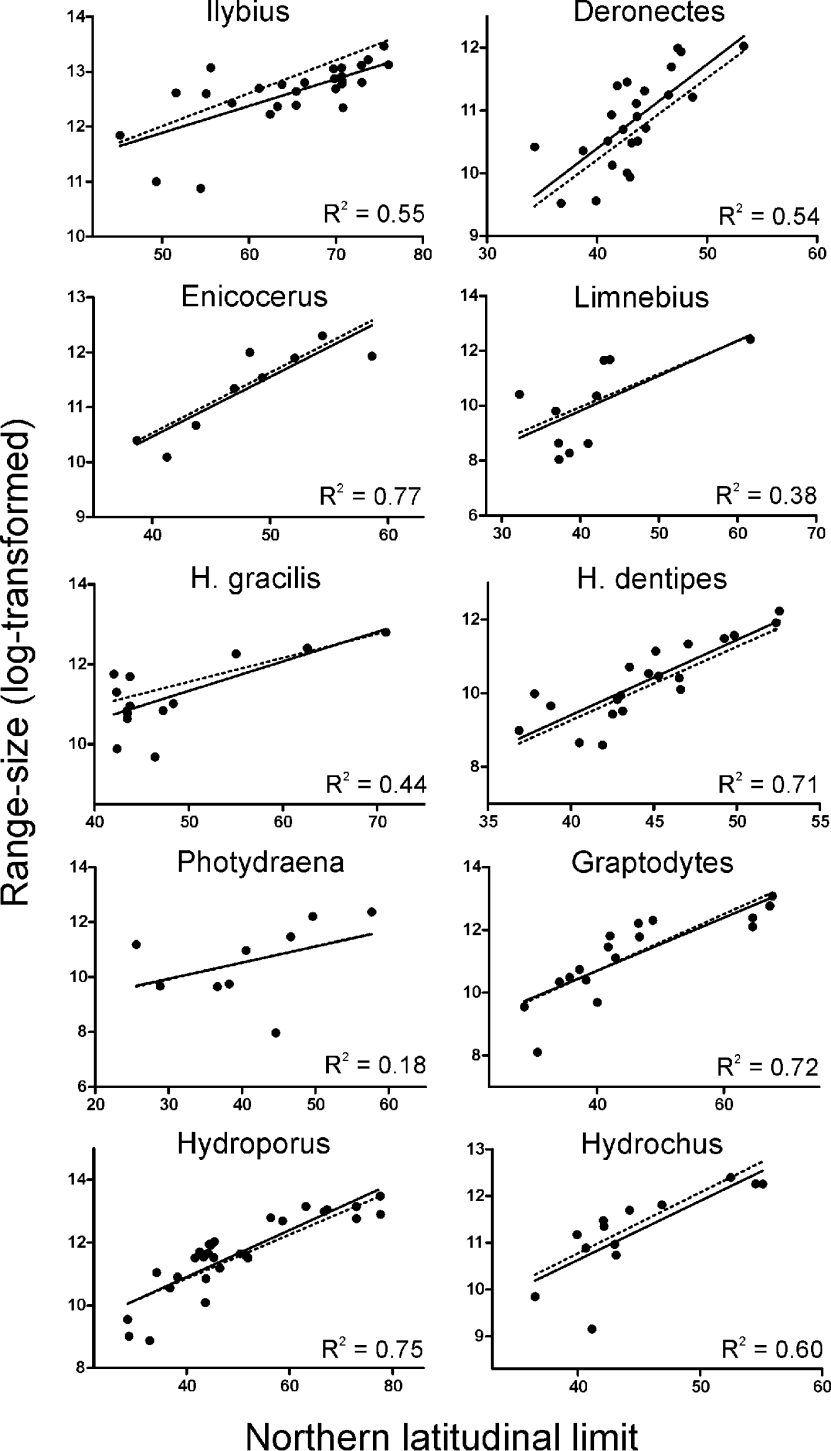
**Northern latitudinal limit against range size**. Influence of the northern latitudinal limit on geographic range size (log-transformed). Raw data points and standard least squares regression line (solid line), without phylogenetic control, are showed for illustrative purposes. The coefficient of determination and the regression line (dotted line) as calculated using Phylogenetic Generalized Least Squares are also provided.

After phylogenetic correction, range size was significantly and positively correlated with maximum latitude and longitude for most of the lineages, with the only exceptions of *Limnebius *(marginally significant) and *Phothydraena *(Table 4; see also Figure 1). These positive correlations remained significant for maximum latitude after Bonferroni correction for multiple tests. Maximum latitude and longitude had also the highest correlation values for most of the lineages. The latitude and longitude of the centroid were correlated with the size of geographic range in four and five of the ten lineages respectively: *Enicocerus*, *Hydraena dentipes*, *Graptodytes *and *Hydroporus*, plus *Ilybius *in the case of latitude of the centroid (Table 4). Minimum longitude and latitude were only significantly correlated with range size in *Ilybius *and *Hydrochus *respectively, in both cases with a negative correlation (Table 4). Range size in *Phothydraena *was not significantly correlated with any of the range proprieties studied, although minimum latitude was marginally significant (Table 4).

### Age and area

**Figure 2:**
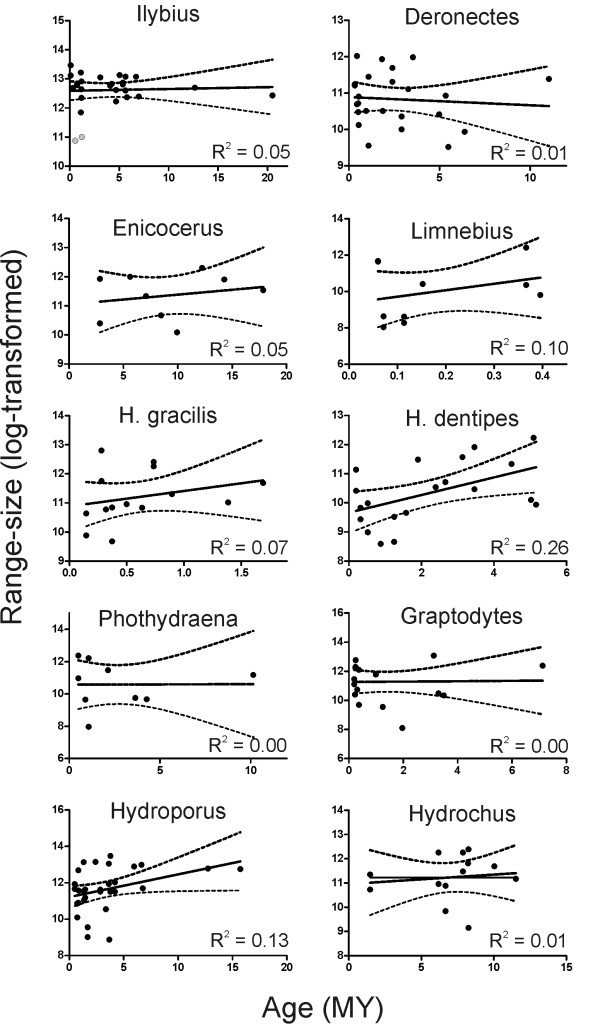
**Range size against species age**. Plots of geographic range size against species age (Myr). The coefficient of determination is provided. With the single exception of *H. dentipes*, slope was not significantly different of 0 in all the studied lineages. The regression line and the 95% confidence interval are also displayed. Grey dots are outliers.

The preferred model for the plot of global geographic range size against species age was in all cases a straight line, i.e. quadratic regression did not provide a significantly better fit to the data than did linear regression (Figure 2; see also Additional file 2, Table S2). The general tendency was to increase range size with evolutionary age, but with the sole exception of the *H. dentipes *lineage (with a significant positive relationship) the values of the slopes of the lines that best predicted range-size from species age were not significantly different from zero (Additional file 1, Table S2), indicating a non significant increase or decline of range size with age.

### Multiple regression models

We assessed the relative importance of the phylogenetic vs. ecological and geographic factors in determining the size of the geographical range through the use of multiple regression models. The percentage of variance explained was in general very high, ranging from ca. 60% to more than 98% (Table 5).

**Table 5:** Stepwise multiple regression models explaining range size

Lineage	*R*^2^	F	*P*	Variables	β	t	*P*
*Ilybius*	75.5	23.66	0.000	maxLat	0.38	3.16	0.004
				PVR24	-0.50	-4.39	0.000
				PVR20	0.35	3.09	0.005
*Deronectes*	63.2	18.03	0.000	maxLat	0.70	5.26	0.000
				PVR19	0.31	2.34	0.030
*Enicocerus*	98.6	121.15	0.000	maxLat	0.52	6.89	0.001
				maxLon	0.47	7.83	0.001
				PVR4	-0.18	-2.83	0.036
*Limnebius*	75.9	11.01	0.007	maxLat	0.55	2.96	0.021
				PVR5	-0.60	-3.18	0.016
*H. gracilis*	69.2	12.35	0.002	maxLat	0.58	3.33	0.007
				PVR1	-0.45	-2.56	0.027
*H. dentipes*	72.3	46.96	0.000	maxLat	0.85	6.85	0.000
*Phothydraena*	81.8	13.49	0.006	minLat	-0.51	-2.89	0.028
				Habitat1	0.69	3.94	0.008
*Graptodytes*	88.8	36.89	0.000	maxLat	0.68	7.06	0.000
				PVR8	-0.32	-3.36	0.005
				Habitat0	-0.26	-2.75	0.016
*Hydroporus*	91.7	69.29	0.000	maxLat	0.70	10.93	0.000
				PVR4	-0.36	-5.77	0.000
				PVR7	-0.17	-2.91	0.008
				PVR21	0.19	3.15	0.004
*Hydrochus*	78.6	18.31	0.000	maxLat	0.59	3.72	0.004
				PVR8	0.46	2.90	0.016

Summary table for the stepwise multiple regression models explaining range size in the different lineages. We show for each model the explained variance (*R*^2 ^in percentage) and its significance (*F *and *P *values). For each variable included in the model, the fitted standardized regression coefficient (β) and its corresponding significance (t and P values) are shown. Variable codes as in Table 2. PVR# refers to phylogenetic vectors used in PVR approach.

For most of the lineages, the maximum latitudinal limit of the geographic range was the most influential variable explaining range size (Table 5). The only exception was *Phothydraena*, for which we used minimum latitude in the model, according to the results of the PGLS analyses (see above).

Although phylogenetic effects had often less influence than biogeographic factors, they were usually retained in the models, and for some lineages were the most important (*Ilybius*, *Limnebius *and *H. gracilis*). Habitat preference was retained in the model for *Phothydraena *(in which was the main range-size determinant) and *Graptodytes*, but not in *Hydroporus *or *Hydrochus*, in which both biogeographic and phylogenetic factors were included in the model. Species' age was not retained in any of the models.

## Discussion

Range-size heritability has sparked an intense debate in the literature in the last years, although no clear conclusions have been drawn, partly because of the variability in the methods employed and the quality of the data used [12,13,67,68] (see [69] for a review). If geographic range sizes are determined by life-history, ecological or physiological characters, it may be expected to find some degree of phylogenetic signal through the cladogenetic process [7,70,71]. Evidence of low phylogenetic signal in range size has been reported in a number of previous studies for different taxonomic groups [14,16,25,29,72-74], the conclusion being that range size is an extremely labile trait. However, as showed by Pigot *et al*. [75], low phylogenetic signal cannot be taken as strong evidence for the lability of geographic ranges. In our case, both the randomization test [15] and the PVR method [35] showed some positive significant phylogenetic signal for several lineages. This phylogenetic signal was relatively weak when compared with other range properties, but still strong enough to be kept as a relevant factor in the multiple regression models (see below). The low taxonomic level of the studied phylogenies could contribute to the lack of significance in the randomization tests of phylogenetic signal for some of the groups [69], as well as the low number of species in some of them (computer simulations demonstrate that this test requires approximately 20 species to achieve a statistical power of 80%, [17]). Nevertheless, the values of the *K *statistic do not depend on sample size, and it is considered a valid descriptive statistic of the amount of phylogenetic signal even for small data sets [17]. The PVR method has a good performance with small phylogenies [76], and in consequence our results from this approach could be more reliable.

The geographic location of the species had the strongest phylogenetic signal of all the variables tested. With the question of the heritability of the size of geographic ranges monopolizing all the attention in the literature, the role of phylogenetic constraints on other attributes of species' ranges, such as geographic position, have remained nearly unexplored (but see [73]). Our results show that, in general, the geographic position of species' ranges display a stronger phylogenetic signal than their size. This is what would be expected under a vicariant mode of speciation in which the ancestral range is split almost randomly (hence partly erasing the phylogenetic signal of range size), but the resulting species maintain the geographic centroid of their ranges through time, so that range movements do not erase completely the geographic signal of speciation. This would justify the use of the present distribution of species to infer speciation processes (e.g., [46,77]), contrary to the view that rapid changes to species geographic ranges effectively eliminate any relationship between the geography of speciation and contemporary locations of geographic ranges [78]. In the case of water beetles, a review of the direct evidence provided by Quaternary remains also support a general pattern of range stability through the last Glacial cycle, contrary to the extended view of generalized major range shifts due to climatic change [79].

When biogeographic, phylogenetic and ecological factors were combined to explain range size differences, the northern limit of the geographic range was generally the main determinant of geographic range size. In a number of terrestrial groups range sizes are known to strongly increase with latitude in the Palearctic and Nearctic above 40°-50°N [26], in agreement with Rapoport's rule (see [80] for a review). In the Western Palearctic, widespread species tend to have a central and north European distribution, and among the water beetles in these areas there are few, if any, species with restricted distributions [32]. In the same way, there are many examples of water beetle lineages including narrow endemics in which the widespread species have the southern limit of their ranges at the edge of the southern peninsulas.

The strong role of geographic location in determining range size can be grounded in different lines of argument. From an ecological perspective, latitudinal/longitudinal gradients can represent a particular case of the more general relationship between the niche breadth of a species and the size of its geographic range (e.g., [2,21,81-83]). Climatic changes and the drastic changes in ecological conditions were specially dramatic in northern latitudes of the Palearctic region, and might have operated as ecological filters [84], with only those species displaying broad ecological niches (and consequently wide ranges) being able to persist in northern regions or re-colonize northern areas from southern refugia after the glaciations. In the studied lineages, maximum latitude was usually highly correlated with niche breadth (Additional file 1, Table S3), showing that those species reaching more northern latitudes display broader ecological niches. Species with narrow niches would have remained restricted to southern areas, less affected by the climatic changes. This is valid also for longitude, with species reaching the more continental parts of Eurasia being more affected by climatic changes. The absence of fossil remains of southern species of aquatic Coleoptera among the abundant central and northern European Quaternary records [79] would support this view, as well as the recognition of the Mediterranean peninsulas as an area of endemism, not as a source of postglacial colonisation (e.g., [85]).

The Western Palearctic has strong ecological constraints in the south and the west (the seas and oceans), which might result in most species having a distribution centre towards the east or the north. The larger range size of the species with a more northern and eastern distribution could thus be the result of a geometric constraint. The species with the largest ranges necessarily include the largest available surfaces, i.e. from central Europe to the east. Any species expanding its range to cover the biogeographical area of the lineages included here will end up with a distribution centroid in the north-east, as species with large distributions approaching the size of a bounded domain are constrained to have their centroid near the centre of the domain [86]. The general negative correlation between minimum latitude and range size (Table 2) is compatible with this geometric effect, showing that widespread species also expand their ranges to the south. But this geometric constraint cannot be the sole explanation for the patterns we found, as this would not explain the phylogenetic signal for both range size and geographical position. The strong positive relationship between maximum latitude and range size shows the asymmetry of the range expansions, with an origin in southern refugia, as also found for European land snails [87]. A more uniform distribution of the ancestral ranges may result in a similar position of the final centroids of the species with expanding ranges, but not in a strong positive relationship between maximum latitude and range size.

Two additional biogeographic factors emerged as highly correlated with range size, the spatial extent and the number of biomes in which species are found. If species can expand their distribution more easily within than across biogeographic boundaries, then species found in biogeographic biomes with a large spatial extent should have larger range sizes than species found in small biomes ([26,88-90]; see [14,25] for examples of application in range-size analyses). The number of biomes can be related with niche breadth differences, since some species are restricted to one (or few) biomes due to habitat specificity while others are able to expand easily their distribution across biomes (Additional file 2), although in this case results are confounded by the unavoidable circularity of the relationship between range size and number of biomes. The heterogeneity of biomes is certainly not uniform over the whole continent, with more climatic and ecological variety in the south associated with the main mountain ranges and the influence of the Mediterranean.

Habitat type was also positively correlated with range-size in those lineages with both lotic and lentic species (the only exception was the genus *Hydrochus*), showing that in the same lineage, and after accounting for possible phylogenetic effects, species inhabiting lentic water bodies display larger distributional ranges than those inhabiting lotic ones, with species inhabiting both types of environments with intermediate range sizes. This is in agreement with previous studies across multiple lineages of freshwater invertebrates, which have shown that lotic species have on average smaller geographical ranges than the lentic species [22,23]. Although most of species included here are winged, there is no information about the flying capacity across species within each one of the lineages, so direct measures of dispersal ability are not available. The differences in spatial and temporal persistence between lotic and lentic habitats (small lentic water bodies tend to fill with sediment over a time period of decades or centuries, while rivers and streams persist over geologically defined time periods) have been postulated as resulting in consistent differences in dispersal strategies and colonization abilities between species living in both types of aquatic environments (see [24] for an overview), providing a surrogate measure of dispersal ability. Since colonization rates depend not only on dispersal abilities, but also on the geographic configuration of habitats, a potential confounding factor could be the differential distribution of suitable habitat between lotic and lentic environments (e.g., a contrasting degree of spatial clustering or a spatial correlation of habitat availability with latitude). Nevertheless, different recent studies have consistently provided evidence against differences in habitat availability, lending further support to the hypothesis that lentic species have a higher propensity for dispersal than lotic species [91-93]. Although dispersal abilities are among the more commonly cited potential determinants of a species' range (see e.g., [94]), this relation has rarely been assessed correcting for phylogeny.

We did not find evidence to support a clear pattern of range size change over time in water beetle lineages. The relationship between age *vs*. range-size plots suggested that species' time since divergence is positively correlated with geographic range size, in agreement with the "range and area" model [28], but the values of the slopes of the lines that best predicted range-size from species age were for most groups not significantly different from zero. Thus, our results are compatible with a "stasis" [15] or an idiosyncratic model, were there is no reason to expect a directional change in geographic range size through time. In any case, the amount of variance in geographic range size explained by phylogenetic age was generally low, as shown by *r*^2 ^values. The lack of a general pattern of the changes in geographic range size over evolutionary time is a common result across a wide range of clades (e.g., [15,29,31,95]. The variability of the type and quality of data used and analyses performed has been viewed as a possible explanation for this lack of consistency [29,95], but our results, using different lineages and a common methodology and dataset, proved to be equally variable and clade-specific, pointing to a true lack of relationship between age and area, despite the uncertainties in the sampling and the delimitation of the ranges (see below).

Other factors not considered in this study may be of relevance in determining geographic range sizes, such as thermal tolerance, body size, population abundance or colonization and extinction dynamics [3]. Similarly, we are aware that our analyses could be weakened by incomplete taxon sampling in some groups and uncertainties in the estimated geographic range sizes. Uncertainties associated to phylogenies are another possible source of error, as missing or extinct taxa would result in the overestimation of the phylogenetic age of the related species. Despite these obvious limitations, the consistency of the results and the high percentage of variance explained by the factors included (between ca. 60 and 98%, Table 5) allows to draw firm conclusions applicable to a wide range of phylogenetically independent groups of Coleoptera.

## Conclusions

Our findings show that range size of a species is shaped by an interplay of geographic and ecological factors, with a phylogenetic component affecting both of them. The understanding of the factors that determine the size and geographical location of the distributional range of species is fundamental to the study of the origin and assemblage of the current biota. Our results show that for this purpose the most relevant data may be the phylogenetic history of the species and its geographical location, in agreement with results from some previous studies (e.g. [14,25]).

## Authors' contributions

Both authors designed and performed research, carried out data compilation and analyses, and participated in preparation of the manuscript. Both authors read and approved the final manuscript.

## Supplementary Material

Additional file 1 **Data used in the study**. Taxa included in the different studied lineages with data on geographic range properties and ecological attributes. Accession numbers of the sequences are also indicated. Click here for file

Additional file 2 **Supplementary tables**. Tables S1-S3. Click here for file

Additional file 3 **Ultrametric trees for the different lineages**. Numbers indicate node support: above nodes, Bayesian posterior probabilities (if above 0.5); below nodes, bootstrap support values from Maximum Likelihood analysis (if above 50%). Click here for file

## References

[B1] Williams CB Patterns in the balance of nature 1964 London: Academic Press

[B2] Brown JH Macroecology 1995 Chicago: University of Chicago Press

[B3] Gaston KJ The structure and dynamics of geographic ranges 2003 New York: Oxford University Press Inc.

[B4] Rosenzweig ML Species diversity in space and time 1995 Cambridge: Cambridge Univiversity Press

[B5] Geber MA Ecological and Evolutionary Limits to Species Geographic Ranges Am Nat 2011 178 S1 S1 S5 10.1086/661899 21956089

[B6] Stevens GC The Latitudinal Gradient in Geographical Range - How So Many Species Coexist in the Tropics Am Nat 1989 133 2 240 256 10.1086/284913

[B7] Brown JH Stevens GC Kaufman DM The geographic range: Size, shape, boundaries, and internal structure Annu Rev Ecol Syst 1996 27 597 623 10.1146/annurev.ecolsys.27.1.597

[B8] Gaston KJ Species-range-size distributions: Patterns, mechanisms and implications Trends Ecol Evol 1996 11 5 197 201 10.1016/0169-5347(96)10027-6 21237808

[B9] Harvey PH Pagel MD The comparative method in evolutionary biology 1991 Oxford University Press, Oxford

[B10] Freckleton RP Jetz W Space versus phylogeny: disentangling phylogenetic and spatial signals in comparative data Proc R Soc B 2009 276 1654 21 30 10.1098/rspb.2008.0905 18796398 PMC2614254

[B11] Webb TJ Gaston KJ On the heritability of geographic range sizes Am Nat 2003 161 4 553 566 10.1086/368296 12776884

[B12] Hunt G Roy K Jablonski D Species-level heritability reaffirmed: A comment on "On the heritability of geographic range sizes" Am Nat 2005 166 1 129 135 10.1086/430722 15937797

[B13] Webb TJ Gaston KJ Heritability of geographic range sizes revisited: A reply to Hunt et al Am Nat 2005 166 1 136 143 10.1086/430726 29648471

[B14] Machac A Zrzavy J Storch D Range Size Heritability in Carnivora Is Driven by Geographic Constraints Am Nat 2011 177 6 767 779 10.1086/659952 21597253

[B15] Jablonski D Heritability at the Species Level - Analysis of Geographic Ranges of Cretaceous Mollusks Science 1987 238 4825 360 363 10.1126/science.238.4825.360 17837117

[B16] Freckleton RP Harvey PH Pagel M Phylogenetic analysis and comparative data: A test and review of evidence Am Nat 2002 160 6 712 726 10.1086/343873 18707460

[B17] Blomberg SP Garland T Ives AR Testing for phylogenetic signal in comparative data: Behavioral traits are more labile Evolution 2003 57 4 717 745 12778543 10.1111/j.0014-3820.2003.tb00285.x

[B18] Pagel M Inferring the historical patterns of biological evolution Nature 1999 401 6756 877 884 10.1038/44766 10553904

[B19] Gaston KJ Rarity 1993 London: Chapman & Hall

[B20] Kunin WE Gaston KJ The Biology of Rarity - Patterns, Causes and Consequences Trends Ecol Evol 1993 8 8 298 301 10.1016/0169-5347(93)90259-R 21236173

[B21] Brown JH On the Relationship between Abundance and Distribution of Species Am Nat 1984 124 2 255 279 10.1086/284267

[B22] Ribera I Vogler AP Habitat type as a determinant of species range sizes: the example of lotic-lentic differences in aquatic Coleoptera Biol J Linn Soc 2000 71 1 33 52

[B23] Hof C Brandle M Brandl R Lentic odonates have larger and more northern ranges than lotic species J Biogeogr 2006 33 1 63 70 10.1111/j.1365-2699.2005.01358.x

[B24] Ribera I Lancaster J, Briers RA Habitat constraints and the generation of diversity in freshwater macroinvertebrates Aquatic Insects: Challenges to Populations 2008 Wallingford: CAB International Publishing

[B25] Bohning-Gaese K Caprano T van Ewijk K Veith M Range size: Disentangling current traits and phylogenetic and biogeographic factors Am Nat 2006 167 4 555 567 10.1086/501078 16670997

[B26] Gaston KJ Blackburn TM Spicer JI Rapoport's rule: time for an epitaph? Trends Ecol Evol 1998 13 2 70 74 10.1016/S0169-5347(97)01236-6 21238203

[B27] Hawkins BA Diniz-Filho JAF Beyond Rapoport's rule: evaluating range size patterns of New World birds in a two-dimensional framework Global Ecol Biogeogr 2006 15 5 461 469

[B28] Willis JC Age and area: a study in geographical distribution and origin of species 1922 Cambridge: Cambridge University Press

[B29] Jones KE Sechrest W Gittleman JL Purvis A, Gittleman JL, Brooks TM Age and area revisited: identifying global patterns and implications for conservation Conserv Biol Ser 2005 Cambridge: Cambridge University Press 141 165

[B30] Gaston KJ Species-range size distributions: products of speciation, extinction and transformation Phil Trans R Soc B 1998 353 1366 219 230 10.1098/rstb.1998.0204

[B31] Webb TJ Gaston KJ Geographic range size and evolutionary age in birds Proc R Soc Lond B 2000 267 1455 1843 1850 10.1098/rspb.2000.1219 PMC1690757 11052534

[B32] Ribera I Foster GN Vogler AP Does habitat use explain large scale species richness patterns of aquatic beetles in Europe? Ecography 2003 26 2 145 152 10.1034/j.1600-0587.2003.03271.x

[B33] Löbl I Smetana A Catalogue of Palaearctic Coleoptera, Volume 1. Archostemata, Myxophaga Adephaga 2003 Copenhagen: Apollo Books

[B34] Löbl I Smetana A Catalogue of Palearctic Coleoptera. Volume 2. Hydrophiloidea-Staphylinoidea 2004 Copenhagen: Apollo Books

[B35] Hunt T Bergsten J Levkanicova Z Papadopoulou A John OS Wild R Hammond PM Ahrens D Balke M Caterino MS A comprehensive phylogeny of beetles reveals the evolutionary origins of a superradiation Science 2007 318 5858 1913 1916 10.1126/science.1146954 18096805

[B36] Nilsson AN World Catalogue of Insects, volume 3, Dytiscidae 2001 Stenstrup: Apollo Books

[B37] Nilsson AN Holmen M The aquatic Adephaga (Coleoptera) of Fennoscandia and Denmark. II. Dytiscidae Fauna Entomol Scand 1995 32 1 192

[B38] Ribera I Hogan JE Vogler AP Phylogeny of hydradephagan water beetles inferred from 18S rRNA sequences Mol Phylogenet Evol 2002 23 1 43 62 10.1006/mpev.2001.1080 12182402

[B39] Ribera I Faille A A new microphthalmic stygobitic Graptodytes Seidlitz from Morocco, with a molecular phylogeny of the genus (Coleoptera, Dytiscidae) Zootaxa 2010 2641 1 14

[B40] Ribera I Bilton DT Balke M Hendrich L Evolution, mitochondrial DNA phylogeny and systematic position of the Macaronesian endemic Hydrotarsus Falkenstrom (Coleoptera: Dytiscidae) Syst Entomol 2003 28 4 493 508 10.1046/j.1365-3113.2003.00226.x

[B41] Ribera I Castro A Hernando C Ochthebius (Enicocerus) aguilerai sp.n. from central Spain, with a molecular phylogeny of the Western Palaearctic species of Enicocerus (Coleoptera, Hydraenidae) Zootaxa 2010 2351 1 13

[B42] Jäch MA Taxonomic revision of the Palearctic species of the genus Limnebius Leach, 1815 (Coleoptera: Hydraenidae) Koleopterol Rundsch 1993 63 99 187

[B43] Fresneda J Ribera I Revision of the Limnebius nitidus (Marsham) subgroup (Coleoptera: Hydraenidae), with description of two new species and comments on their phylogeny and biogeography Entomol Scand 1998 29 4 395 409 10.1163/187631298X00032

[B44] Hansen M World Catalogue of Insects. Volume 1. Hydraenidae (Coleoptera) 1998 Stenstrup: Apollo Books

[B45] Jäch MA Löbl I, Smetana A Hydraenidae Catalogue of Palaearctic Coleoptera, Volume 2: Hydrophiloidea - Histeroidea - Staphylinoidea 2004 Stenstrup: Apollo Books 102 122

[B46] Ribera I Castro A Díaz-Pazos JA Garrido J Izquierdo A Jäch MA Valladares LF The geography of speciation in narrow range endemics of the "Haenydra" lineage (Coleoptera, Hydraenidae, *Hydraena*) J Biogeogr 2011 38 502 516 10.1111/j.1365-2699.2010.02417.x

[B47] Jäch MA Beutel R Díaz JA Kodada J Subgeneric classification, description of head structures, and world check list of *Hydraena *Kugelann (Insecta: Coleoptera: Hydraenidae) Ann Naturhist Mus 2000 102 B 177 258

[B48] Hansen M World Cataloque of Insects. Volume 2. Hydrophiloidea (Coleoptera) 1999 Stenstrup: Apollo Books

[B49] Hidalgo-Galiana A Ribera I Late Miocene diversification of the genus Hydrochus (Coleoptera, Hydrochidae) in the west Mediterranean area J Biogeogr 2011 59 377 385 10.1016/j.ympev.2011.01.018 21354316

[B50] Stamatakis A Hoover P Rougemont J A Rapid Bootstrap Algorithm for the RAxML Web Servers Syst Biol 2008 57 5 758 771 10.1080/10635150802429642 18853362

[B51] Miyata T Katoh K Misawa K Kuma K MAFFT: a novel method for rapid multiple sequence alignment based on fast Fourier transform Nucleic Acids Res 2002 30 14 3059 3066 10.1093/nar/gkf436 12136088 PMC135756

[B52] Drummond AJ Rambaut A BEAST: Bayesian evolutionary analysis by sampling trees Bmc Evol Biol 2007 7 10.1186/1471-2148-7-214 PMC2247476 17996036

[B53] Papadopoulou A Anastasiou I Vogler AP Revisiting the Insect Mitochondrial Molecular Clock: The Mid-Aegean Trench Calibration Mol Biol Evol 2010 27 7 1659 1672 10.1093/molbev/msq051 20167609

[B54] Brower AVZ Desalle R Practical and Theoretical Considerations for Choice of a DNA-Sequence Region in Insect Molecular Systematics, with a Short Review of Published Studies Using Nuclear Gene Regions Ann Entomol Soc Am 1994 87 6 702 716

[B55] Brower AVZ Rapid Morphological Radiation and Convergence among Races of the Butterfly Heliconius-Erato Inferred from Patterns of Mitochondrial-DNA Evolution Proc Natl Acad Sci USA 1994 91 14 6491 6495 10.1073/pnas.91.14.6491 8022810 PMC44228

[B56] Queney P Liste taxonomique des Coléoptères "aquatiques" de la Fauna de France Le Coléoptériste 2004 7 3 3 3921700548

[B57] Sánchez-Fernández D Lobo JM Abellán P Ribera I Millán A Bias in freshwater biodiversity sampling: the case of Iberian water beetles Divers Distrib 2008 14 5 754 762 10.1111/j.1472-4642.2008.00474.x

[B58] Kembel SW Cowan PD Helmus MR Cornwell WK Morlon H Ackerly DD Blomberg SP Webb CO Picante: R tools for integrating phylogenies and ecology Bioinformatics 2010 26 11 1463 1464 10.1093/bioinformatics/btq166 20395285

[B59] Diniz JAF De Sant'ana CER Bini LM An eigenvector method for estimating phylogenetic inertia Evolution 1998 52 5 1247 1262 10.2307/2411294 28565378

[B60] Morand S Poulin R Phylogenies, the comparative method and parasite evolutionary ecology Adv Parasit 2003 54 281 302 10.1016/s0065-308x(03)54006-4 14711088

[B61] Harmon LJ Weir JT Brock CD Glor RE Challenger W GEIGER: investigating evolutionary radiations Bioinformatics 2008 24 1 129 131 10.1093/bioinformatics/btm538 18006550

[B62] Martins EP Hansen TF Phylogenies and the comparative method: A general approach to incorporating phylogenetic information into the analysis of interspecific data Am Nat 1997 149 4 646 667 10.1086/286013

[B63] Martins EP Computer programs for the statistical analysis of comparative data. Distributed by the author at http://compare.bio.indiana.edu/. 2004 Bloomington IN: Department of Biology, Indiana University

[B64] Felsenstein J Phylogenies and the Comparative Method Am Nat 1985 125 1 1 15 10.1086/284325

[B65] Paradis E Claude J Strimmer K APE: Analyses of Phylogenetics and Evolution in R language Bioinformatics 2004 20 2 289 290 10.1093/bioinformatics/btg412 14734327

[B66] Montgomery DC Peck EA Introduction to Regression Analysis 1982 New York: Wiley

[B67] Mouillot D Gaston KJ Geographical range size heritability: what do neutral models with different modes of speciation predict? Global Ecol Biogeogr 2007 16 3 367 380 10.1111/j.1466-8238.2007.00292.x

[B68] Jablonski D Hunt G Larval ecology, geographic range, and species survivorship in Cretaceous mollusks: Organismic versus species-level explanations Am Nat 2006 168 4 556 564 10.1086/507994 17004227

[B69] Waldron A Null models of geographic range size evolution reaffirm its heritability Am Nat 2007 170 2 221 231 10.1086/518963 17874373

[B70] Lawton JH Range, Population Abundance and Conservation Trends Ecol Evol 1993 8 11 409 413 10.1016/0169-5347(93)90043-O 21236213

[B71] Emlet RB Developmental Mode and Species Geographic Range in Regular Sea-Urchins (Echinodermata, Echinoidea) Evolution 1995 49 3 476 489 10.2307/2410272 28565076

[B72] Diniz JAF Torres NM Phylogenetic comparative methods and the geographic range size - body size relationship in new world terrestrial carnivora Evol Ecol 2002 16 4 351 367 10.1023/A:1020210321776

[B73] Nabout JC Terribile LC Bini LM Diniz-Filho JAF Phylogenetic autocorrelation and heritability of geographic range size, shape and position of fiddler crabs, genus Uca (Crustacea, Decapoda) J Zool Syst Evol Res 2010 48 2 102 108 10.1111/j.1439-0469.2009.00531.x

[B74] Blackburn TM Jones KE Cassey P Losin N The influence of spatial resolution on macroecological patterns of range size variation: a case study using parrots (Aves: Psittaciformes) of the world J Biogeogr 2004 31 2 285 293 10.1046/j.0305-0270.2003.01018.x

[B75] Pigot AL Phillimore AB Owens IPF Orme CDL The Shape and Temporal Dynamics of Phylogenetic Trees Arising from Geographic Speciation Syst Biol 2010 59 6 660 673 10.1093/sysbio/syq058 20952757

[B76] Martins EP Diniz JAF Housworth EA Adaptive constraints and the phylogenetic comparative method: A computer simulation test Evolution 2002 56 1 1 13 11913655

[B77] Barraclough TG Vogler AP Detecting the geographical pattern of speciation from species-level phylogenies Am Nat 2000 155 4 419 434 10.1086/303332 10753072

[B78] Losos JB Glor RE Phylogenetic comparative methods and the geography of speciation Trends Ecol Evol 2003 18 5 220 227 10.1016/S0169-5347(03)00037-5

[B79] Abellan P Benetti CJ Angus RB Ribera I A review of Quaternary range shifts in European aquatic Coleoptera Global Ecol Biogeogr 2011 20 1 87 100 10.1111/j.1466-8238.2010.00572.x

[B80] Gaston KJ Chown SL Why Rapoport's rule does not generalise Oikos 1999 84 2 309 312 10.2307/3546727

[B81] Ruggiero A Hawkins BA Mapping macroecology Global Ecol Biogeogr 2006 15 5 433 437

[B82] Morin X Chuine I Niche breadth, competitive strength and range size of tree species: a trade-off based framework to understand species distribution Ecol Lett 2006 9 2 185 195 10.1111/j.1461-0248.2005.00864.x 16958884

[B83] Addo-Bediako A Chown SL Gaston KJ Thermal tolerance, climatic variability and latitude Proc R Soc Lond B 2000 267 1445 739 745 10.1098/rspb.2000.1065 PMC1690610 10819141

[B84] Dynesius M Jansson R Evolutionary consequences of changes in species' geographical distributions driven by Milankovitch climate oscillations Proc Natl Acad Sci USA 2000 97 16 9115 9120 10.1073/pnas.97.16.9115 10922067 PMC16831

[B85] Bilton DT Mirol PM Mascheretti S Fredga K Zima J Searle JB Mediterranean Europe as an area of endemism for small mammals rather than a source for northwards postglacial colonization Proc R Soc Lond B 1998 265 1402 1219 1226 10.1098/rspb.1998.0423 PMC1689182 9699314

[B86] Lyons SK Willig MR Latitudinal patterns of range size: Methodological concerns and empirical evaluations for New World bats and marsupials Oikos 1997 79 3 568 580 10.2307/3546901

[B87] Hausdorf B Latitudinal and altitudinal diversity patterns and Rapoport effects in north-west European land snails and their causes Biol J Linn Soc 2006 87 2 309 323 10.1111/j.1095-8312.2006.00580.x

[B88] Roy K Jablonski D Valentine JW Eastern Pacific Molluscan Provinces and Latitudinal Diversity Gradient - No Evidence for Rapoports Rule Proc Natl Acad Sci USA 1994 91 19 8871 8874 10.1073/pnas.91.19.8871 11607494 PMC44708

[B89] Smith FDM May RM Harvey PH Geographical Ranges of Australian Mammals J Anim Ecol 1994 63 2 441 450 10.2307/5561

[B90] Ribera I Biogeography and conservation of Iberian water beetles Biol Conserv 2000 92 2 131 150 10.1016/S0006-3207(99)00048-8

[B91] Dehling DM Hof C Brandle M Brandl R Habitat availability does not explain the species richness patterns of European lentic and lotic freshwater animals J Biogeogr 2010 37 10 1919 1926

[B92] Hof C Brandle M Brandl R Latitudinal variation of diversity in European freshwater animals is not concordant across habitat types Global Ecol Biogeogr 2008 17 4 539 546 10.1111/j.1466-8238.2008.00394.x

[B93] Abellan P Millan A Ribera I Parallel habitat-driven differences in the phylogeographical structure of two independent lineages of Mediterranean saline water beetles Mol Ecol 2009 18 18 3885 3902 10.1111/j.1365-294X.2009.04319.x 19702753

[B94] Lester SE Ruttenberg BI Gaines SD Kinlan BP The relationship between dispersal ability and geographic range size Ecol Lett 2007 10 8 745 758 10.1111/j.1461-0248.2007.01070.x 17594430

[B95] Paul JR Morton C Taylor CM Tonsor SJ Evolutionary Time for Dispersal Limits the Extent but Not the Occupancy of Species' Potential Ranges in the Tropical Plant Genus Psychotria (Rubiaceae) Am Nat 2009 173 2 188 199 10.1086/595762 19140770

