# Supplementary material for: Geographic location and phylogeny are the main determinants of the size of the geographical range in aquatic beetles
Source: BMC Evol Biol. 2011 Nov 28;11:344. doi: 10.1186/1471-2148-11-344 (PMC3247920; doi:10.1186/1471-2148-11-344)
Supplement: Additional file 2 — Supplementary tables. Tables S1-S3. [file 1471-2148-11-344-S2.PDF]

Supplementary tables

Table A1. List of the primers used.

Gene	Name	Sense	Sequence	Reference
cox1	Jerry (M202)	F	CAACATTTATTTTGATTTTTTGG	1
	Pat (M70)	R	TCCA(A)TGCACTAATCTGCCATATTA	1
	Chy	F	T(A/T)GTAGCCCA(T/C)TTTCATTA(T/C)GT	2
	Tom	R	AC(A/G)TAATGAAA(A/G)TGGGCTAC(T/A)A	2
	Tom-2	R	A(A/G)GGGAATCATTGAATAAA(A/T)CC	2
cyb	CB3	F	GAGGAGCAACTGTAATTACTAA	3
	CB4	R	AAAAGAAA(AG)TATCATTCAGGTTGAAT	3
rrnL-nad1	16saR (M14)	F	CGCCTGTTTA(A/T)CAAAAACAT	1
	16Sa	R	ATGTTTTTGTTAAACAGGCG	1
	16Sb	R	CCGGTCTGAACTCAGATCATGT	1
	16SAlf1	R	GCATCACAAAAAGGCTGAGG	4
	ND1A (M223)	R	GGTCCCTTACGAATTTGAATATATCCT	1
	16Sbi	F	ACATGATCTGAGTTCAAACCGG	1
	FawND1	R	TAGAATTAGAAGATCAACCAGC	1
rrnS	12S ai	F	AAACTAGGATTAGATACCCTATTAT	1
	12S bi	R	AAGAGCGACGGGCGATGTGT	1
SSU	5'	F	GACAACCTGGTTGATCCTGCCAGT	5
	b5.0	R	TAACCGCAACAACTTTAAT	5
LSU	Ka	F	ACACGGACCAAGGAGTCTAGCATG	2
	Kb	R	CGTCCTGCTGTCTTAAGTTAC	2

[1] Simon C., Frati F., Beckenbach A., Crespi B., Liu H. & Flook P. 1994: Evolution, weighting, and phylogenetic utility of mitochondrial gene-sequences and a compilation of conserved polymerase chain-reaction primers. Ann. ent. Soc. Am. 87: 651-701.

[2] Ribera I., Fresneda J., Bucur R., Izquierdo A., Vogler A.P., Salgado J.M. & Cieslak A. 2010: Ancient origin of a Western Mediterranean radiation of subterranean beetles. BMC Evolutionary Biology 10: 29.

[3] Barraclough T.G., Hogan J.E. & Vogler A.P. 1999: Testing whether ecological factors promote cladogenesis in a group of tiger beetles (Coleoptera : Cicindelidae). Proc R Soc Lond B 266(1423):1061-1067.

[4] Vogler A.P., Desalle R., Assmann T., Knisley C.B. & Schultz T.D. 1993: Molecular population genetics of the endangered tiger beetle Cicindela dorsalis (Coleoptera, Cicindelidae). Ann ent Soc Am 86: 142-152.

[5] Shull V.L., Vogler A.P., Baker M.D., Maddison D.R., Hammond P.M. 2001: Sequence alignment of 18S ribosomal RNA and the basal relationships of Adephagan beetles: evidence for monophyly of aquatic families and the placement of Trachypachidae. Syst. Biol., 50: 945-969.

Table A2. Results of age *versus* range-size regressions.

Lineage	R^2	slope	slope different 0?	linear vs. quadratic
Ilybius	0.007	0.011	$F_{1,25} = 0.18$, $p = 0.676$	$F_{1,24} = 1.12$, $p = 0.302$
Deronectes	0.006	-0.022	$F_{1,22} = 0.14$, $p = 0.710$	$F_{1,21} = 0.89$, $p = 0.355$
Enicocerus	0.047	0.034	F1,7 = 0.35 p= 0.573	$F_{1,6} = 0.11$, $p = 0.756$
Limnebius	0.1	3.545	$F_{1,8} = 0.89$, $p = 0.373$	$F_{1,7} = 0.43$, $p = 0.532$
H. gracilis	0.072	0.528	$F_{1,12}=0.93$, $p = 0.353$	$F_{1,11} = 0,70$, $p = 0.421$
H. dentipes	0.258	0.303	$F_{1,18} = 6.27$, $p = 0.022$	$F_{1,17} = 0.34$, $p = 0.570$
Phothydraena	0.000	0.003	$F_{1,7} = 0.00$, $p = 0.986$	$F_{1,6} = 0.81$, $p = 0.404$
Graptodytes	0.000	0.013	$F_{1,16}=0.01$, $p = 0.942$	$F_{1,15} = 2.47$, $p = 0.137$
Hydroporus	0.129	0.125	$F_{1,28} = 4.18$, $p = 0.050$	$F_{1,27} = 0.26$, $p = 0.613$
Hydrochus	0.014	0.039	$F_{1,11} = 0.16$, $p = 0.701$	$F_{1,10} = 0.00$, $p = 0.989$

Table A3. Results of phylogenetic comparative tests for associations between the northern latitudinal limit of geographic species' range and biogeographical and ecological variables using Phylogenetic Generalized Least Squares.

		maxLon	minLon	minLat	LonC	LatC	aveB	nB	Hab
Ilybius	α	15.5	15.5	15.5	15.5	15.5	15.5	15.5	
	r	0.20	-0.35	0.26	-0.14	0.91	0.14	0.44	--
	P	0.254	0.101	0.068	0.589	**0.000***	0.520	0.076	
Deronectes	α	15.5	15.5	15.5	15.5	15.5	15.5	15.5	
	r	0.31	-0.09	0.27	0.15	0.8	0.54	0.57	--
	P	0.094	0.957	0.153	0.341	**0.000***	**0.004***	**0.004***	
Enicocerus	α	15.5	15.5	15.5	15.5	2.69	15.5	15.5	
	r	0.58	0.22	0.19	0.47	0.96	0.64	0.72	--
	P	0.105	0.571	0.631	0.207	**0.000***	0.063	**0.028**	
Limnebius	α	0.98	0.81	0.70	0.56	5.97	0.64	15.5	
	r	0.812	-0.171	0.26	0.59	0.96	-0.04	0.79	--
	P	**0.005**	0.680	0.467	0.072	**0.000***	0.870	**0.008**	
H. gracilis	α	15.5	2.81	11.17	15.5	1.20	15.5	15.5	
	r	0.57	-0.53	0.18	0.3	0.97	0.33	0.43	--
	P	**0.031**	0.192	0.584	0.280	**0.000***	0.232	0.108	
H. dentipes	α	2.25	0.99	0.80	1.50	15.50	1.44	2.37	
	r	0.76	0.06	0.37	0.53	0.96	0.48	0.77	--
	P	**0.000***	0.469	0.081	**0.004***	**0.000***	**0.008**	**0.000***	
Phothydraena	α	15.5	15.5	15.5	15.5	15.5	15.5	15.5	5.03
	r	0.1	-0.3	0.37	-0.11	0.93	0.14	0.59	0.83
	P	0.860	0.411	0.276	0.730	**0.000***	0.796	0.100	**0.014**
Graptodytes	α	6.89	2.07	6.70	5.79	15.50	3.34	6.06	2.90
	r	0.70	-0.42	0.34	0.4	0.87	0.58	0.720	0.05
	P	**0.000***	0.212	0.054	**0.041**	**0.000***	**0.009**	**0.000***	0.871
Hydroporus	α	15.50	2.13	9.66	15.50	15.50	6.67	15.50	6.10
	r	0.82	-0.38	0.36	0.6	0.95	0.59	0.79	0.59
	P	**0.000***	0.138	**0.038**	**0.001**	**0.000***	**0.000***	**0.000***	**0.000***
Hydrochus	α	4.24	2.58	2.88	3.42	6.00	15.50	4.50	2.36
	r	0.61	-0.27	-0.34	0.23	0.86	0.55	0.51	0.51
	P	**0.024**	0.422	0.229	0.421	**0.000***	**0.050**	0.069	0.102

The maximum likelihood estimate of evolutionary constraint (α) is provided, and parameter estimates given that maximum likelihood. Significant correlations are indicated with boldface type. Asterisk indicates significant p-values after Bonferroni correction for multiple tests ($p < 0.005$). Codes as in Table 2; Hab, habitat preference (lotic, lentic or both).
