# Supplementary material for: Geographic location and phylogeny are the main determinants of the size of the geographical range in aquatic beetles
Source: BMC Evol Biol. 2011 Nov 28;11:344. doi: 10.1186/1471-2148-11-344 (PMC3247920; doi:10.1186/1471-2148-11-344)

Ultrametric trees for the different lineages. Numbers indicate node support: above nodes, Bayesian posterior probabilities (if above 0.5); below nodes, bootstrap support values from Maximum Likelihood analysis (if above 50%). Time-scale in millions of years.

Ilybius lineage

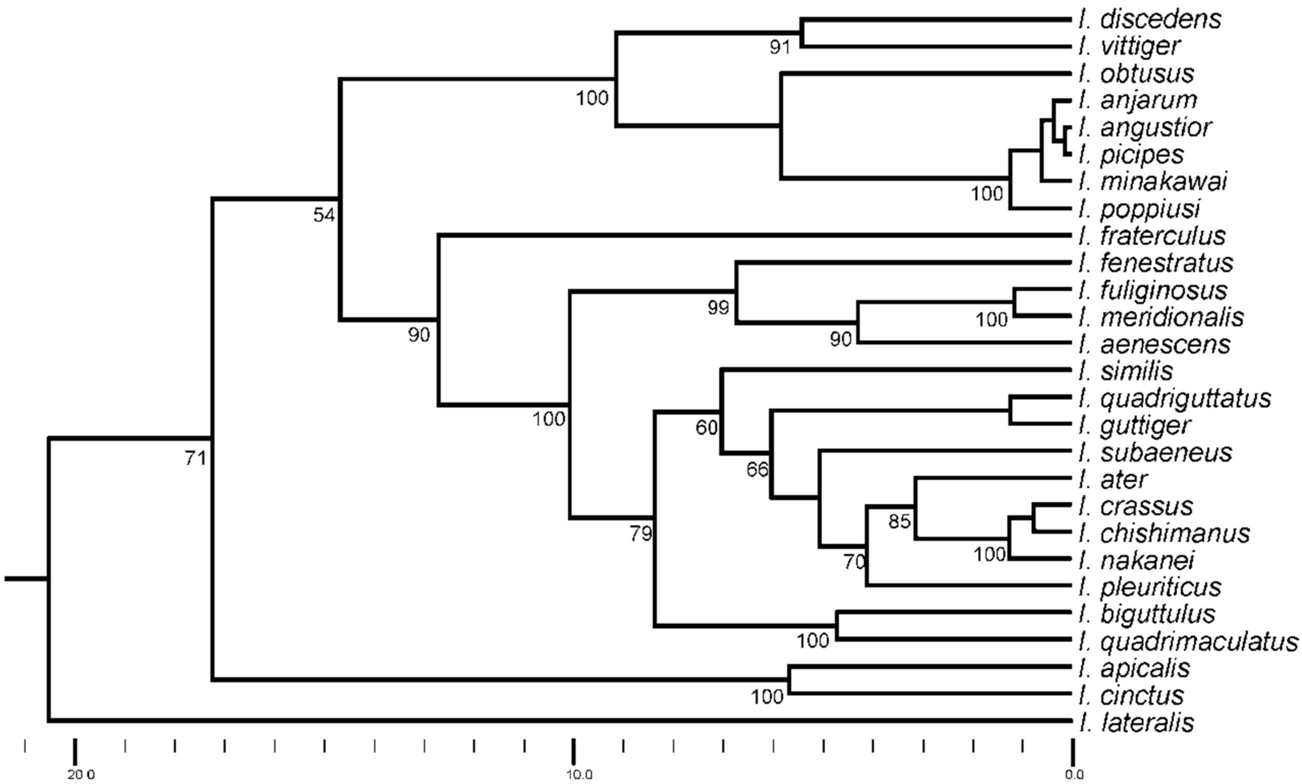

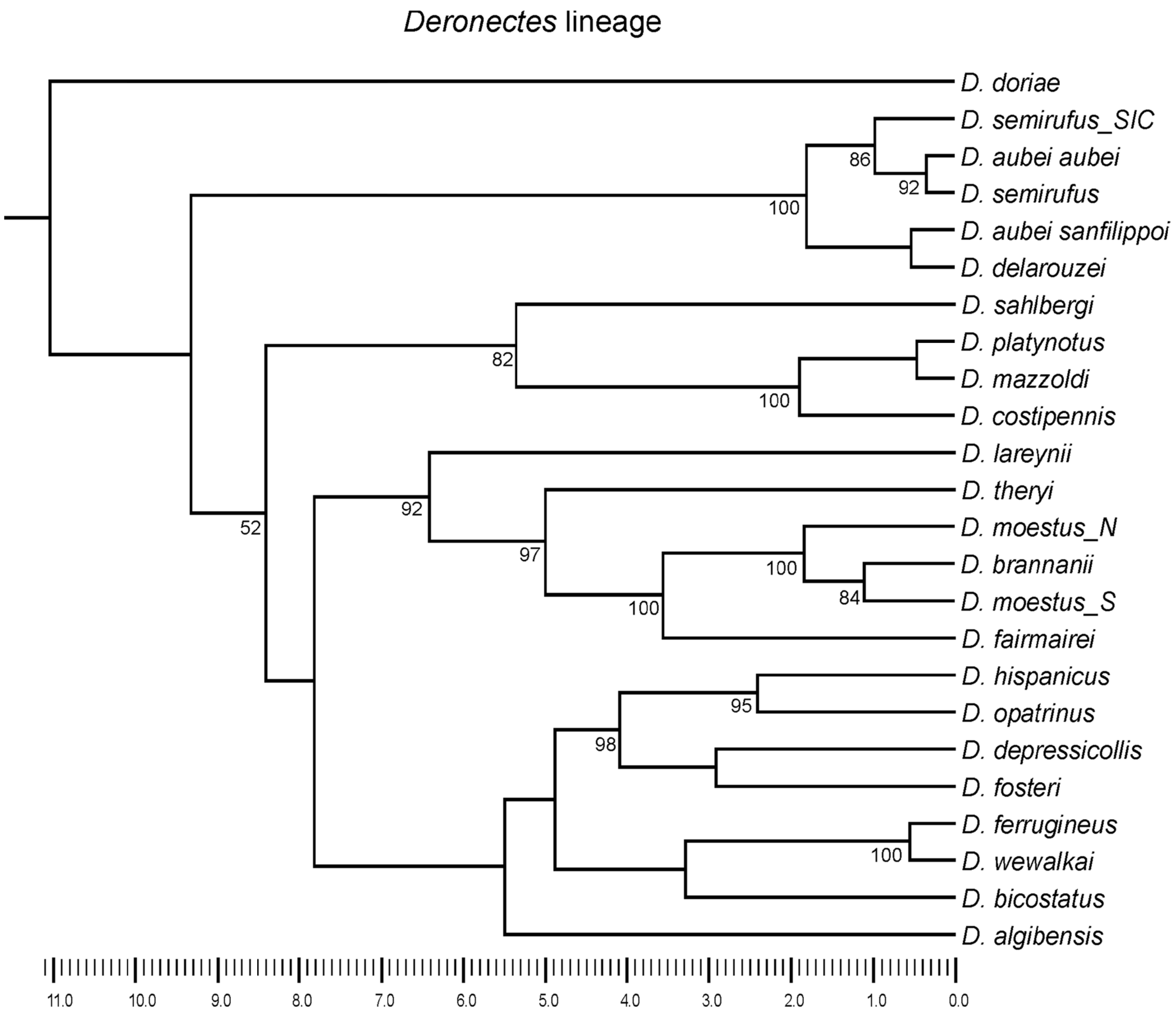

Deronectes lineage
D. doriae
D. semirufus_SIC
D. aubei aubei
D. semirufus
D. aubei sanfilippoi
D. delarouzei
D. sahlbergi
D. platynotus
D. mazzoldi
D. costipennis
D. lareynii
D. theryi
D. moestus_N
D. brannanii
D. moestus_S
D. fairmairei
D. hispanicus
D. opatrinus
D. depressicollis
D. fosteri
D. ferrugineus
D. wewalkai
D. bicostatus
D. algibensis
86
92
100
82
100
52
92
97
100
100
84
95
98
100
11.0 10.0 9.0 8.0 7.0 6.0 5.0 4.0 3.0 2.0 1.0 0.0

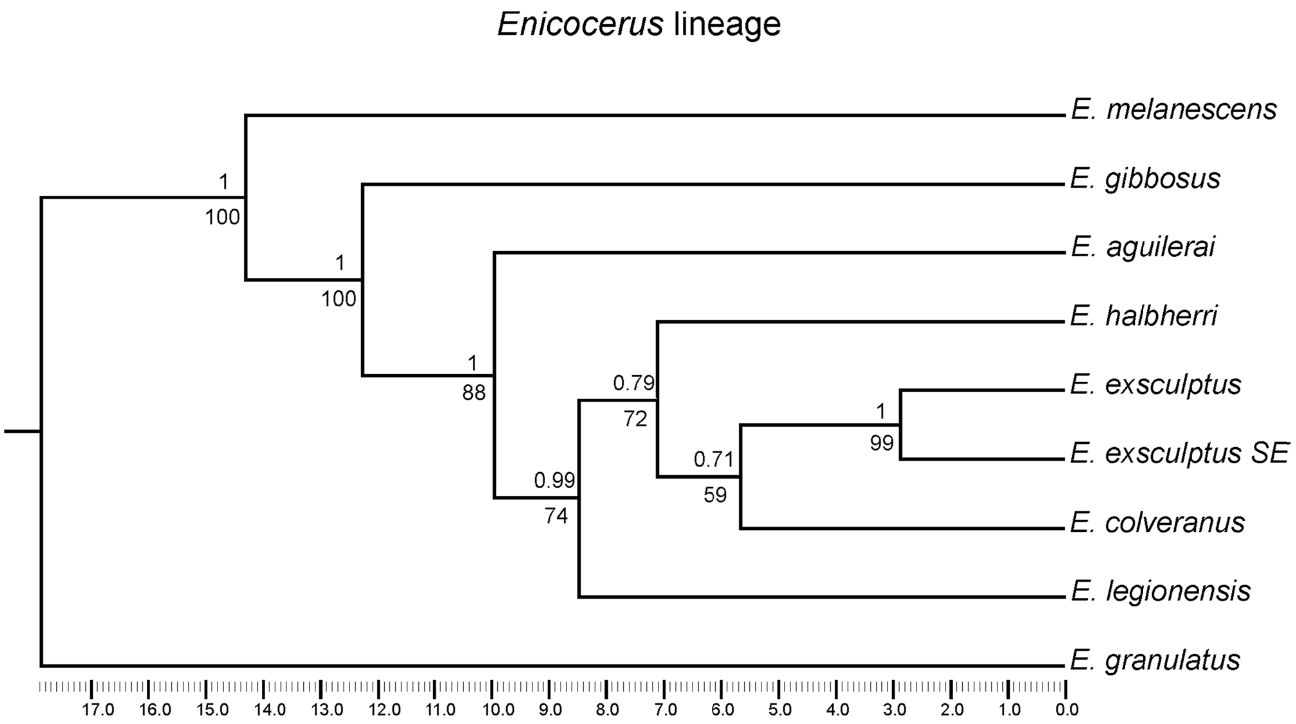

Enicocerus lineage
E. melanescens
E. gibbosus
E. aguilerai
E. halbherri
E. exsculptus
E. exsculptus SE
E. colveranus
E. legionensis
E. granulatus
1
100
1
100
1
88
0.79
72
0.99
74
0.71
59
1
99
17.0 16.0 15.0 14.0 13.0 12.0 11.0 10.0 9.0 8.0 7.0 6.0 5.0 4.0 3.0 2.0 1.0 0.0

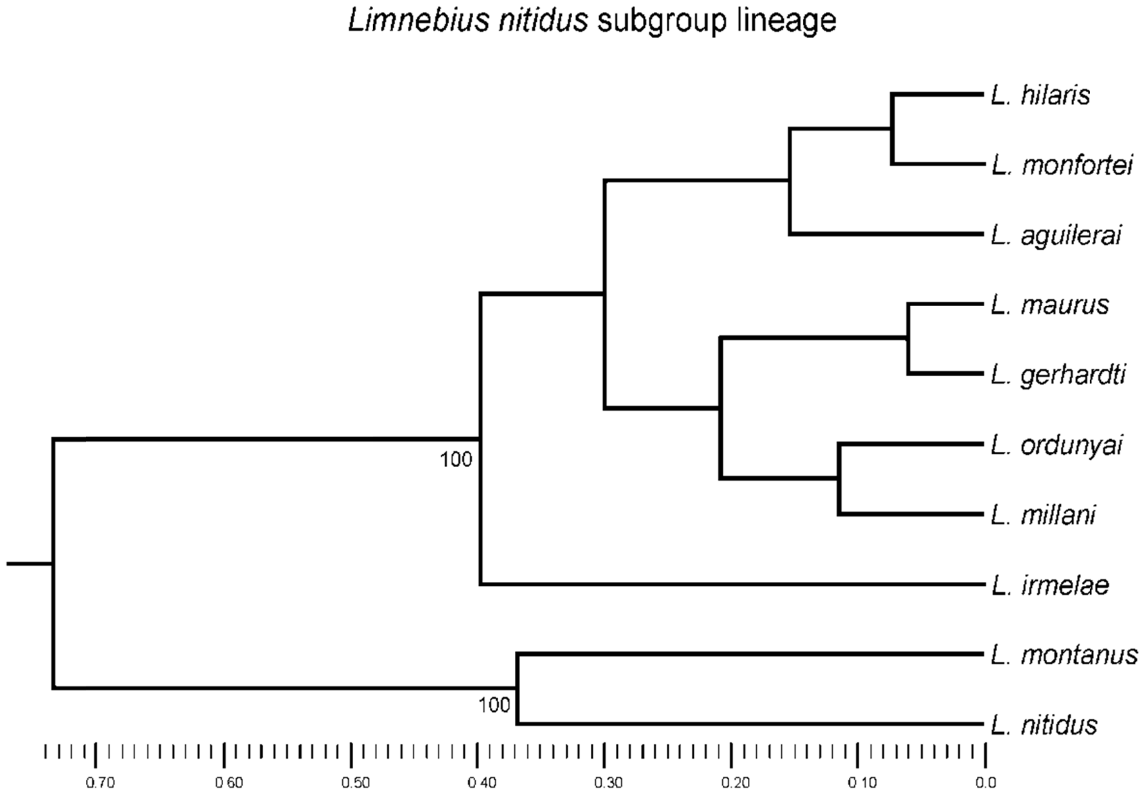

Limnebius nitidus subgroup lineage
L. hilaris
L. monfortei
L. aguilerai
L. maurus
L. gerhardti
L. ordunyai
L. millani
L. irmelae
L. montanus
L. nitidus
100
100
0.70 0.60 0.50 0.40 0.30 0.20 0.10 0.0

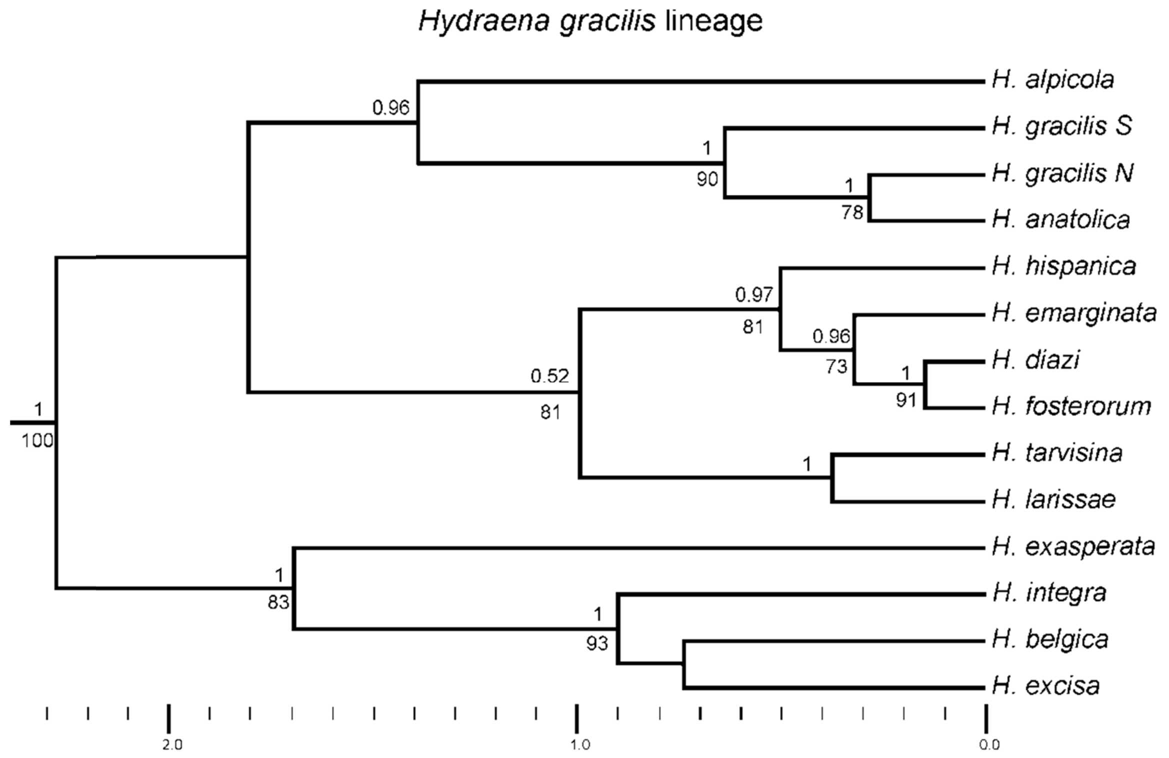
Hydraena gracilis lineage
H. alpicola
H. gracilis S
H. gracilis N
H. anatolica
H. hispanica
H. emarginata
H. diazi
H. fosterorum
H. tarvisina
H. larissae
H. exasperata
H. integra
H. belgica
H. excisa
0.96
1
90
1
78
0.97
81
0.96
73
1
91
0.52
81
1
1
100
1
83
1
93
2.0
1.0
0.0

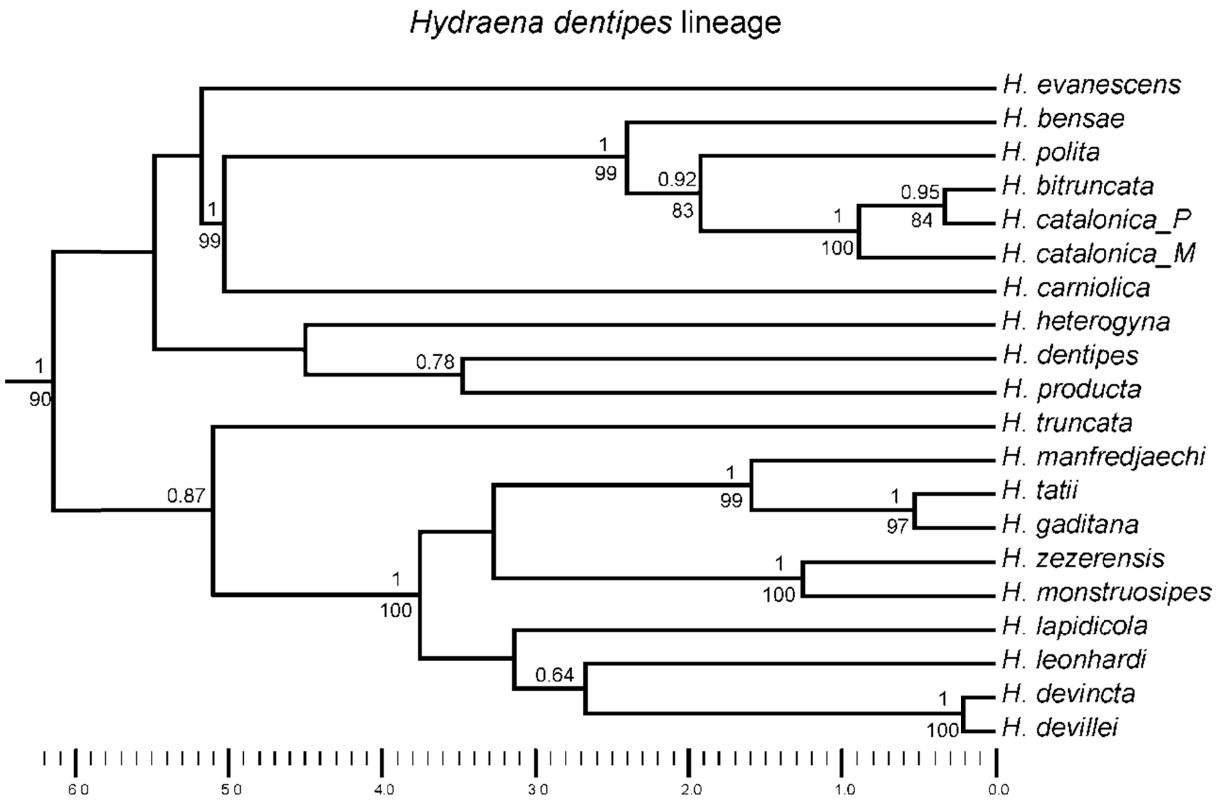
Hydraena dentipes lineage
H. evanescens
H. bensae
H. polita
H. bitruncata
H. catalonica_P
H. catalonica_M
H. carniolica
H. heterogyna
H. dentipes
H. producta
H. truncata
H. manfredjaechi
H. tatii
H. gaditana
H. zezerensis
H. monstruosipes
H. lapidicola
H. leonhardi
H. devincta
H. devillei
1
99
0.92
83
0.95
84
1
100
1
99
0.78
1
90
0.87
1
99
1
97
1
100
1
100
0.64
1
100
6.0
5.0
4.0
3.0
2.0
1.0
0.0

“Phothydraena” lineage

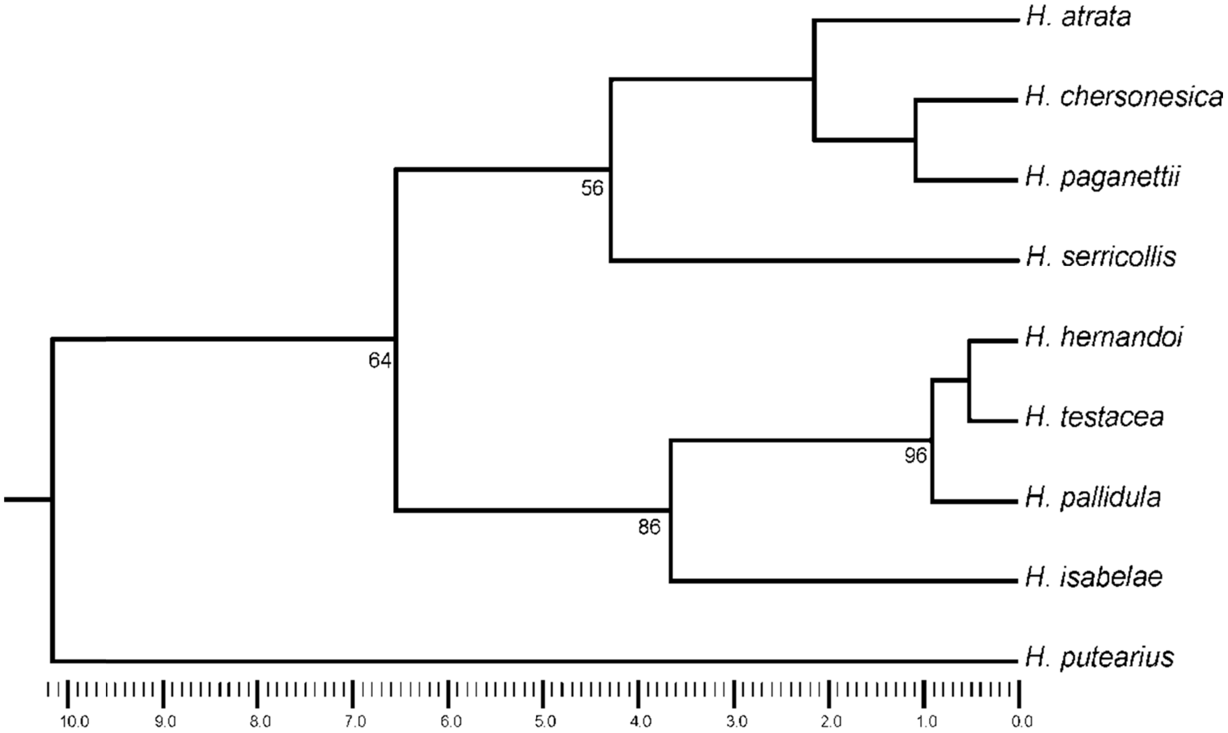

Graptodytes lineage

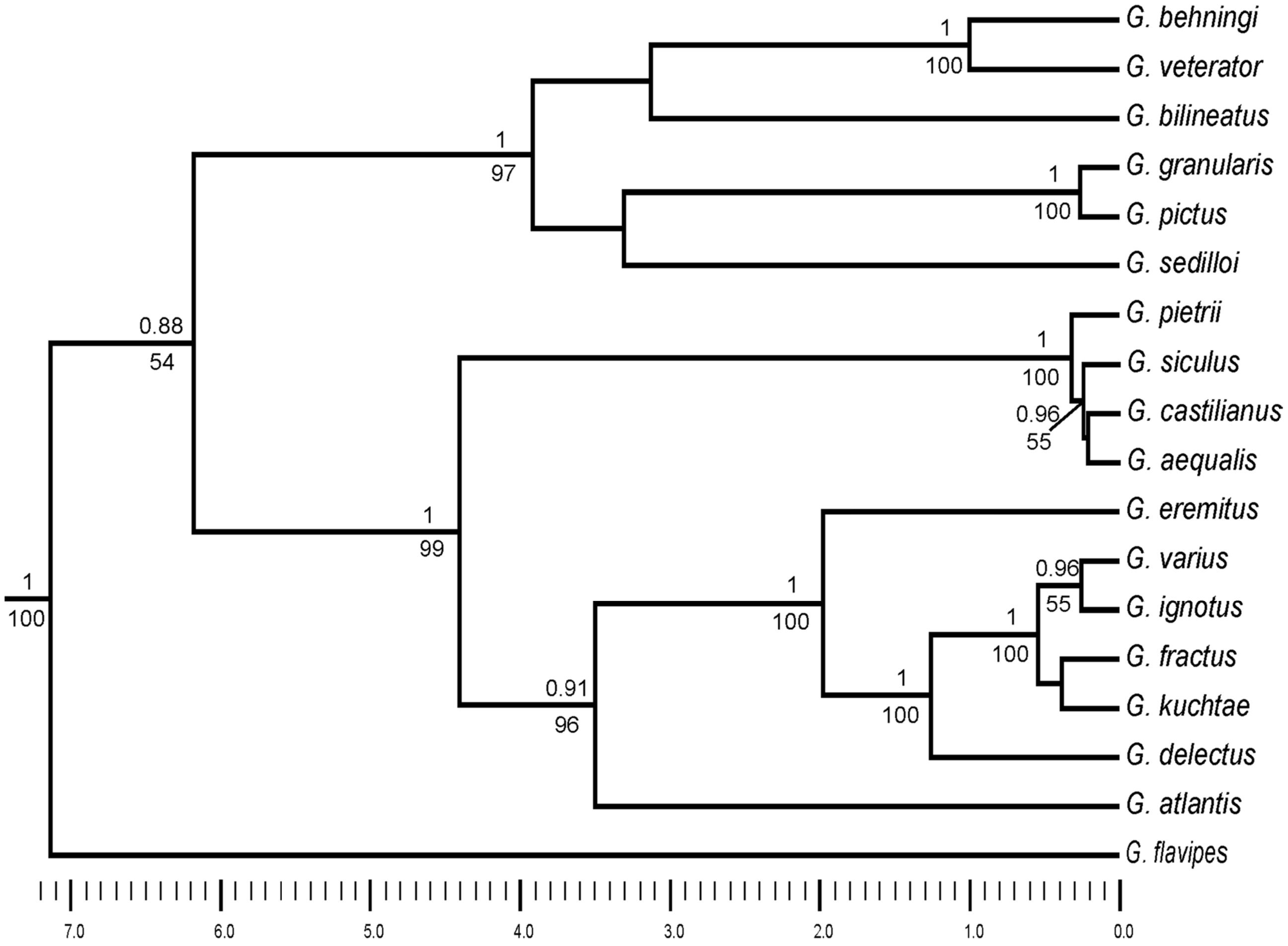

Hydroporus lineage

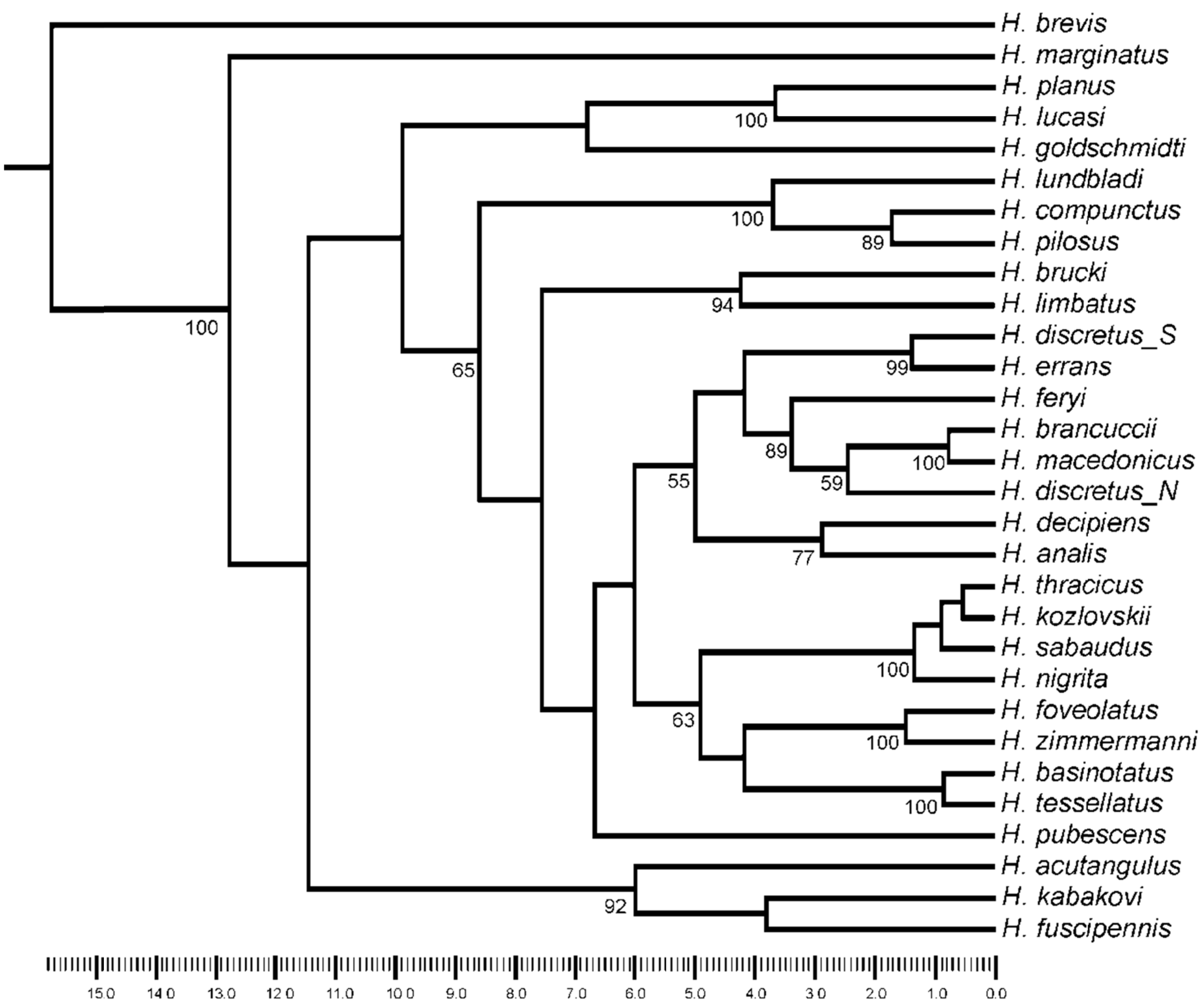

Hydrochus lineage

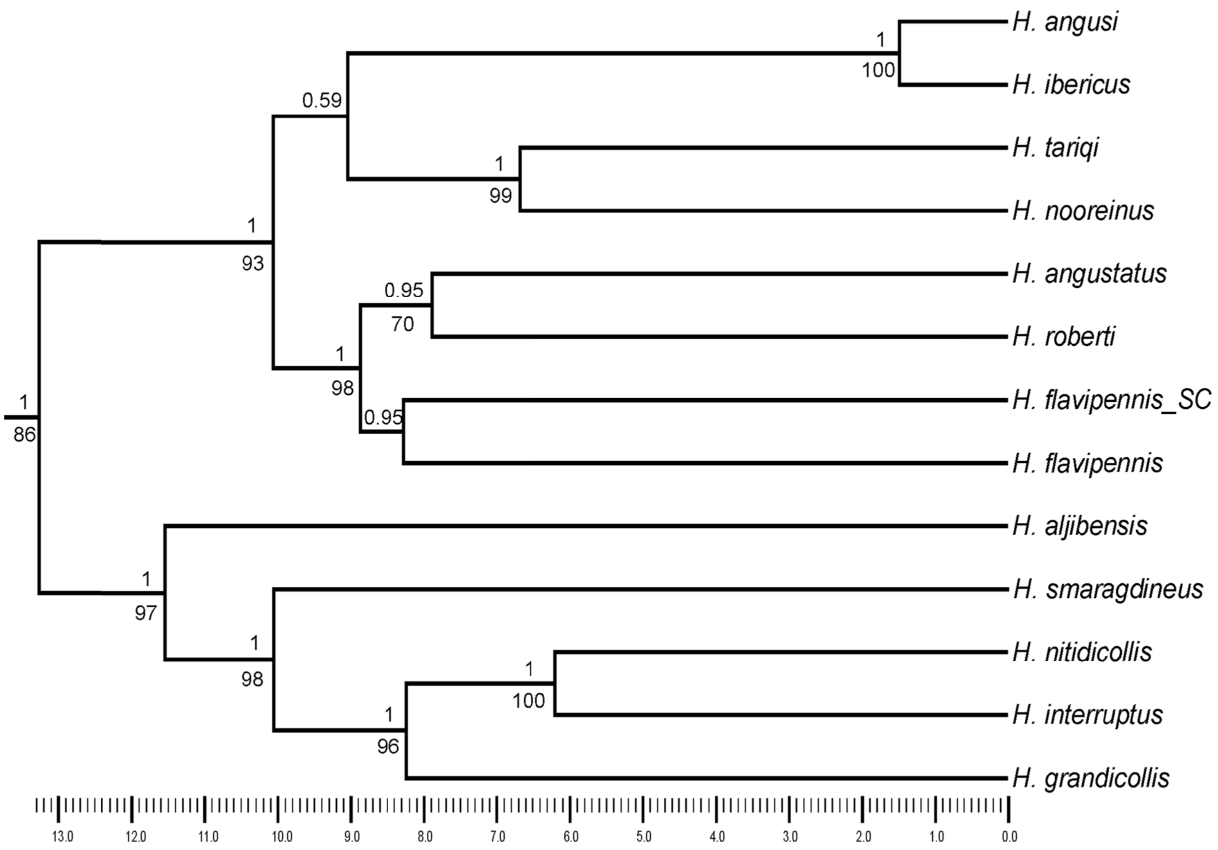

Supplement: Additional file 3 — Ultrametric trees for the different lineages. Numbers indicate node support: above nodes, Bayesian posterior probabilities (if above 0.5); below nodes, bootstrap support values from Maximum Likelihood analysis (if above 50%). [file 1471-2148-11-344-S3.PDF]
